# Xylan Degradation in the Halotolerant Bacterium *Bacillus altitudinis* relies on glycosidic hydrolases from families 11 and 30

**DOI:** 10.1021/acs.jafc.5c06247

**Published:** 2025-10-16

**Authors:** Alessandro Marchetti, Marco Orlando, Stefania Digiovanni, Christos Christakis, Vasileios Tsopanakis, Nikolaos Arapitsas, Ioannis V. Pavlidis, Panagiotis Sarris, Marco Mangiagalli, Marina Lotti

**Affiliations:** 1 Department of Biotechnology and Biosciences, 9305 University of Milano-Bicocca, Piazza della Scienza 2, Milano 20126, Italy; 2 Department of Biology, 37777 University of Crete, Voutes University Campus, Heraklion 70013, Greece; 3 Institute of Molecular Biology and Biotechnology, Fountation for Research and Technology Hellas, Nik. Plastira Str., Vasilika Vouton, Heraklion 70013, Greece; 4 Department of Chemistry, 37777 University of Crete, Voutes University Campus, Heraklion 70013, Greece; 5 School of Life Sciences, University of Exeter, Stocker Road, Exeter EX4 4QD, United Kingdom

**Keywords:** glycoside hydrolases (GH), *Bacillus altitudinis* SRL571, salt-tolerant enzymes, endophytic bacteria, polysaccharides degradation

## Abstract

The breakdown of xylan, a major hemicellulose component, involves multiple xylanases.*Bacillus altitudinis* SRL571, a halotolerant endophytic bacterium, utilizes glucuronoxylan and xylose as its sole carbon and energy sources. Genome analysis revealed two sequences encoding putative secreted xylanolytic glycoside hydrolases: one from family 11 (BaGH11) and another from family 30, subfamily 8 (BaGH30). These genes are located in two distinct operons involved in xylan and xylose catabolism, a genomic configuration unique to this strain. Both enzymes are salt-tolerant and act as endoxylanases: BaGH11 releases mainly short-chain xylooligosaccharides (e.g., xylobiose) while BaGH30 produces medium-chain xylooligosaccharides. BaGH11 and BaGH30 act synergistically to hydrolyze glucuronoxylan into xylose and xylobiose, which are subsequently imported into cells via putative sugar transporters. This study elucidates the biocatalytic basis of xylan degradation in a halotolerant bacterium and highlights the importance of complementary enzyme activities for effective biomass degradation in saline environments.

## Graphical abstract

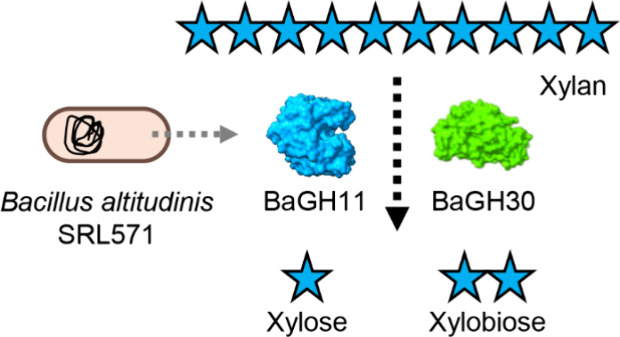

## Introduction

Coastal areas are the interfaces between terrestrial and marine environments, where glycans typical of both environments can be found.^1^ These polysaccharides may originate from plants or algae that are transported by waves or tides.^2,3^ Xylan is a heteropolysaccharide consisting of a linear backbone of D-xylopyranose with β-1,4 glycosidic bonds, often branched with methyl glucuronic acids (MeGA) and/or other sugars such as α-arabinose, which is a the main component of hemicellulose in terrestrial plants and seeds, and is also synthesized by red and green algae.^4–6^

The degradation of xylan into monosaccharides requires the synergistic action of multiple hydrolytic enzymes, including β-xylanases, β-xylosidases, and α-l-arabinofuranosidases.^7–9^ Xylanases are endoenzymes that cleave the β-(1,4) glycosidic bonds of the backbone, resulting in the generation of short xylooligosaccharides (XOS), while β-xylosidases remove xylose from the nonreducing end of XOS.^10,11^ According to the carbohydrate-active enzyme (CAZy) database, glycosyl hydrolase (GH) families 5, 7, 8, 10, 11, 30, and 43 are classified as β-xylanases, while GH families 3, 11, 30, 39, 43, and 52 are classified as β-xylosidases.^12^ Xylanolytic enzymes have been identified mainly in fungi and bacteria, including several *Bacillus* sp.^13,14^ In particular, *B. subtilis*sp. 168 was shown to efficiently hydrolyze hemicellulose from hardwood through a xylanolytic system composed of two secreted β-xylanases belonging to the GH11 (XynA) and GH30 (XynC) families, and a secreted α-L-arabinofuranosidase of the GH43 (XynD) family.^15,16^ This xylanolytic system produces xylobiose and small XOS from xylan and arabinoxylan, which are then internalized by the XynP transporter and metabolized by the bacterium via other accessory enzymes, including a GH43_11 family exoxylanase (XynB), a xylose isomerase (XylA) and a xylulose kinase (XylB).^17–20^ All the genes encoding for these enzymes, except *xynA*, are located in three different operons, *xynDC*, *xynPB* and *xylAB*. The last two operons are under the control of the XynR repressor, which is sensitive to intracellular xylose levels.^15,17,19,20^

Coastal areas are characterized by remarkable biodiversity and are populated by halophyte plants^21^ which have evolved various adaptive strategies to survive in high-salt environments, including the production of compatible solutes to increase cytoplasmic osmotic pressure, and the excretion of sodium ions from cells.^22^ Another adaptive mechanism developed by halophytes to mitigate the detrimental effects of high salinity is the symbiosis with halophilic/halotolerant endophytic bacteria.^23–25^ These symbiotic bacteria colonize various plant tissues, such as leaves, roots, seeds, and flowers,^26,27^ playing a crucial role in salt tolerance, and in the distribution and release of metabolites, phytohormones, and nutrients within plants.^28^ Halophilic endophytic bacteria are indeed a relevant source of secreted hydrolytic enzymes, including xylanases.^29^ Overall, halophilic enzymes are characterized by their ability to maintain structure and function under conditions of low water activity and limited solvation, which triggers denaturation or aggregation of nonhalophilic proteins. The mechanisms of salt adaptation in these enzymes have been studied mainly in the Archea and include a protein surface enriched in negatively charged residues that form a stable salt ion and/or hydration shell, and a reduction in hydrophobic surface sites.^9,29^

This study investigates the xylan-degrading enzymatic setup of *Bacillus altitudinis* SRL571, an endophytic halotolerant bacterium isolated from the inner leaf tissues of *Cakile maritima*, a plant native of Ierapetra beach on the island of Crete. *C. maritima* is known for its ability to tolerate salinity fluctuations and to accumulate NaCl in its leaves. In this context, *B. altitudinis* SRL571 is part of an endophytic bacterial consortium that includes members of the genera *Oceanobacillus* and *Staphylococcus*.^30^ Genome analysis identified two secreted xylanases, which were produced in recombinant form and tested for their activity. These enzymes were characterized as endoxylanases belonging to the GH family 11 (BaGH11) and the GH family 30, subfamily 8 (BaGH30). The study demonstrates that the combined use of these two enzymes can effectively hydrolyze xylan, suggesting potential applications of these halotolerant enzymes for hemicellulose degradation in biotechnological processes.

## Materials and Methods

### Materials

Glucuronoxylan from beechwood (Megazyme code: P-XYLNBE), arabinoxylan from wheat flour (Megazyme code: P-WAXYL) and xyloglucan from tamarind seed (Megazyme code: P-XYGLN) were purchased from Megazyme (International Bray, Ireland). 3,5 dinitrosalicylic acid (DNS), NaCl, ampicillin, Na-pyruvate, Congo Red and xylose were purchased from Merck (Merck, Darmstadt, Germany).

### Growth Conditions

Glucuronoxylan degradation by *B. altitudinis* SRL571 was preliminary investigated using the Congo red assay^31^ on modified R2A medium (yeast extract 0.5 g/L, peptone 0.5 g/L, casamino acids 0.5 g/L, Na-pyruvate 0.3 g/L, KH_2_PO_4_ 0.3 g/L, MgSO_4_ 0.1 g/L) agar plates supplemented with 1 g/L of xylan. The plates were streaked with *B. altitudinis* SRL571 and incubated at 30 °C for 72 h. Subsequently, the plates were flooded with a 0.1% w/v Congo Red solution and incubated at room temperature for 15 min with shaking (60 rpm). The Congo Red was then removed, and the plates were flooded with 1 M NaCl and incubated at room temperature for 15 min with shaking (60 rpm).

Growth in medium containing either xylose or glucuronoxylan as the sole carbon source was assessed in M9 medium in the absence and in the presence of 1 g/L xylose or glucuronoxylan. Precultures were grown in Luria–Bertani broth (tryptone 10 g/L, yeast extract 5 g/L, NaCl 5 g/L) at 30 °C up to OD_600_ ∼ 1, then centrifuged, washed twice with physiological solution and resuspended in M9 medium to OD_600_ ∼ 0.05. Bacterial cultures were incubated at 30 °C with shaking (120 rpm) for 7 days.

Growth kinetics were monitored over time by measuring OD_600_ with a Jasco V-770 UV/NIR spectrophotometer (JASCO Europe, Lecco, Italy). To characterize the glucuronoxylan degradation products, after 48 h of incubation, 2.5 mL of cell culture were centrifuged at 4 °C at 1000 x *g* for 15 min and ultrapure acetonitrile was added to the supernatants to 80% v/v final concentration. Samples were subsequently incubated on ice for 15 min and then centrifuged at 4 °C at 18000 x *g* for 15 min. The clarified supernatant was lyophilized using a SpeedVac SPD120 lyophilizer (ThermoFisher Scientific, US), resuspended in 100 uL of 50% v/v acetonitrile, and analyzed by high-performance liquid chromatography coupled to a single quadrupole mass detector (HPLC-MS) as described in the next paragraphs.

### Bioinformatic Analyses

Genes coding for putative GHs were searched with *hhmscan* from HMMER v3.3.2 ^32^ in the genome of *B. altitudinis* SRL571 (GenBank BioSample ID: SAMN14989801), using the family/subfamily profile hidden Markov models from dbCAN2, with a restrictive E-value of e^–30^.^33^ The domains were annotated using Pfam software.^34^ Signal peptides were predicted using SignalP 6.0.^35^ Enzymes were functionally annotated using BLAST top-hit against the CAZy database (accessed on 01/10/2024). The operon-mapper web server^36^ was employed to identify operons containing the xylanolytic enzymes of *B. altitudinis* SRL571 and from closely related*B. subtilis* *sp.* 168, for which a full genome is available. The 3D models of BaGH11 and BaGH30 were predicted using AF2 ^37^ and ColabFold v.1.5.5 (https://github.com/sokrypton/ColabFold). Structural alignment was performed using DALI^38^ and multiple sequence alignment with Clustal Omega.^39^

### Gene Design, Expression, and Purification of the Recombinant Enzymes

Sequences coding for BaGH11 (Uniprot ID: C8CB65) and BaGH30 (Uniprot ID: A0A653RLR4) without the secretion signal peptide (positions 1–27 and 1–32 for BaGH11 and BaGH30, respectively) were codon-optimized for expression in *Escherichia coli*cells (Genscript, Piscataway, NJ, USA) and cloned *in frame* with a C-terminal 6x His-Tag into pET21 plasmid (EMD, Millipore, Billerica, MA, USA) between *Nde*I and *Xho*I restriction sites. The plasmids were used to transform*E. coli*BL21 (DE3) cells (EMD, Millipore, Billerica, MA, USA). Recombinant enzymes were produced for 24 h at 25 °C in Zym 5052 medium^40^ supplemented with 100 mg/L of ampicillin. Finally, cells were harvested, and proteins were extracted and purified as described in ref 41.

### Standard Xylanase Activity Assay

Unless otherwise specified, the standard xylanase activity assay was performed in a 100 μL final volume containing 0.5 mg/mL of purified enzyme and 5 g/L of glucuronoxylan in an appropriate buffer. Reactions were incubated for 7.5 min at 800 rpm in a thermal shaker (Eppendorf, Hamburg, Germany). The amount of reducing sugar released was quantified using the DNS method.^42^ At the end of incubation, reactions were stopped by adding 400 μL of DNS reagent and heating at 99 °C for 5 min, as described in ref 33. Absorbance was measured at 540 nm using a Jasco V-770 UV/NIR spectrophotometer (JASCO Europe, Lecco, Italy), and sugar concentration was determined against a xylose calibration curve. One unit (U) of xylanase was defined as the amount of enzyme that released one micromole of xylose equivalent per minute.

#### Determination of Optimal pH and Temperature

The optimal catalysis conditions were determined using glucuronoxylan as the substrate. The pH_opt_ was measured in the pH range 3.0–10.0 in Britton–Robinson buffer at temperatures of 55 °C for BaGH11 and 60 °C for BaGH30. *T*_*opt*_ was determined in the temperature range 10–90 °C, at pH 7.0 for BaGH11 and at pH 8.0 for BaGH30.

#### Determination of Substrate Specificity

Substrate specificity was tested using the following polysaccharides: glucuronoxylan from beechwood, wheat arabinoxylan, and xyloglucan from tamarind seeds. Enzyme activity was evaluated using the standard assay under their respective optimal pH and temperature conditions (pH 7.0 and 55 °C for BaGH11 and pH 8.0 and 60 °C for BaGH30).

### Effect of NaCl on the Activity and Stability of BaGH11 and BaGH30

The effect of NaCl on the activity of BaGH11 and BaGH30 was studied using standard xylanase assays with the following modifications. Reactions were incubated in the absence or in the presence of an increasing concentration of NaCl (0–3 M) in PB at pH 7.0 for BaGH11 and pH 8.0 for BaGH30. Samples were incubated for 24 h at 10 °C below *T_opt_* (45 °C for BaGH11, 50 °C for BaGH30).

Thermal denaturation experiments were carried out by monitoring, in the absence or in the presence of increasing concentrations of NaCl, the circular dichroism (CD) signal at 220 nm as a function of temperature in the range of 10–90 °C using a Jasco J815 spectropolarimeter (JASCO Europe, Lecco, Italy). Measurements were performed in a 0.1 cm path length quartz cuvette with a temperature slope of 1 °C/min.

Kinetic stability was assessed by measuring residual activity over time, after incubating purified enzymes at 0.5 mg/mL in PB (pH 7.0 for BaGH11 and pH 8.0 for BaGH30) with and without different concentrations of NaCl, at 45 °C for BaGH11 and 50 °C for BaGH30. Residual activity was determined by using glucuronoxylan as a substrate, and reducing sugars were determined by the DNS assay after 10 min of incubation at *T*_*opt*_. Half-life times (*t*_1/2_) were calculated using a linear regression equation of a semilog plot of relative residual activity versus incubation time. All experiments were performed in triplicate and are reported as mean ± standard deviation.

### Glucuronoxylan Degradation

Enzymatic reactions were performed in ammonium acetate buffer (pH 7.0 for BaGH11 and pH 8.0 for BaGH30), in the presence of 10 g/L glucuronoxylan and 1 mg/mL of each enzyme. The reaction mixtures were incubated in a thermal shaker (Eppendorf, Hamburg, Germany) at 30 °C and 800 rpm for 24 h. These suboptimal temperature conditions offer a good compromise between providing an adequate amount of sugar for HPLC-MS sensitivity and observing the intermediate degradation products. At the end of the incubation period, 200 μL samples were withdrawn, and ultrapure acetonitrile (Carlo Erba, Italy) was added to a final concentration of 80% v/v. Subsequently, the samples were incubated on ice for 15 min and centrifuged at 4 °C at 18000 x *g*. The clarified supernatants were diluted with ultrapure water to a final acetonitrile concentration of 50% v/v and then analyzed by HPLC-MS.

To study the combined effects of the two enzymes, reactions containing 10 g/L of glucuronoxylan were carried out in an ammonium acetate buffer solution at pH 7.0 with 1 mg/mL BaGH30 for 24 h, as previously described. Then, 1 mg/mL BaGH11 was added, and the reaction proceeded for an additional 24 h. After the first and second steps, 200 μL samples were withdrawn, and ultrapure acetonitrile was added to achieve an 80% (v/v) final concentration. Acetonitrile-treated samples were analyzed as described below.

The synergistic effects of BaGH11 and BaGH30 were evaluated by preparing reactions in ammonium acetate buffer at pH 7.5 containing glucuronoxylan (10 g/L) and a molar ratio of BaGH30 and BaGH11 of 1:1, 1:0, and 0:1. The reactions were incubated for 48 h as previously described. Reducing sugars were determined using DNS.

### HPLC-MS Analysis of Glucuronoxylan Degradation Products

The products released from glucuronoxylan by the enzyme treatment were determined using an Autopurification system (Waters, Milford, MA, USA) coupled with an Acquity QDa detector (Waters, Milford, MA, USA). Chromatographic separation was performed with the Waters 2545 binary gradient module on a XBridge BEH Amide Column 4.6 × 150 mm 3.5 μm (Waters, Milford, MA, USA), equipped with a vanguard column, operating at room temperature. The mobile phase consisted of water (A) and acetonitrile, supplemented with 0.1% ammonia (B). Twenty μL of samples and standards were loaded with the Waters 2767 sample manager and elution was performed with the following gradient: linear gradient from 75% B to 45% B in 7 min, 1 min at 45% B and equilibration to initial conditions for 17 min. The flow rate was set at 0.8 mL/min, and mass detection was conducted with an electrospray ionization source operating in negative ion mode. The following molecular masses were used for selected ion monitoring: 149 Da (xylose, 1X), 281 Da (xylobiose, 2X), 413 Da (xylotriose, 3X), 545 Da (xylotetraose, 4X), 809 Da (xylohexaose, 6X), and 1073 Da (8X). As beech xylan also contains few MeGA decorations, the formation of methyl-d-glucuronoxylan (MeGAX) oligosaccharides was also investigated. The capillary and cone voltages were set to 0.8 kV and 5 V, respectively for each compound, except for 8X, requiring a cone voltage of 30 V. Data were acquired with Masslynx software v4.2 (Waters, Milford, MA, USA) and processed using OriginLab software (OriginLab Corporation, Northampton, MA, USA).

## Results

### *B. altitudinis* SRL571 Catabolizes Xylan and Xylose

Degradation of glucuronoxylan by *B. altitudinis* SRL571 cells was preliminarily assessed using the Congo red assay. After 48 h of incubation at 30 °C on xylan-supplemented agar plates, a clear halo was observed around colonies (Figure S1), suggesting the secretion of extracellular xylan-degrading enzymes. Based on these data, cells were grown in shake flasks containing M9 minimal media supplemented with either glucuronoxylan or xylose (1 g/L) as the sole carbon source. *B. altitudinis* SRL571 grew on both substrates, reaching an OD_600_ of 0.25 and 0.2 after 2 (glucuronoxylan) and 3 (xylose) days of incubation at 30 °C (Figure 1 **A, B**). After 2 days of growth in glucuronoxylan-based medium, HPLC-MS analysis of the culture supernatant revealed the presence of xylose (1X), xylobiose (2X) and xylotriose (3X), as well as MeGAX_2_ and MeGAX_3_ oligosaccharides (Figure 1 **C, D**). Taken together, these results suggest that this strain secretes xylanases, triggering the breakdown of glucuronoxylan into xylose and short-chain oligosaccharides.

**Figure 1:**
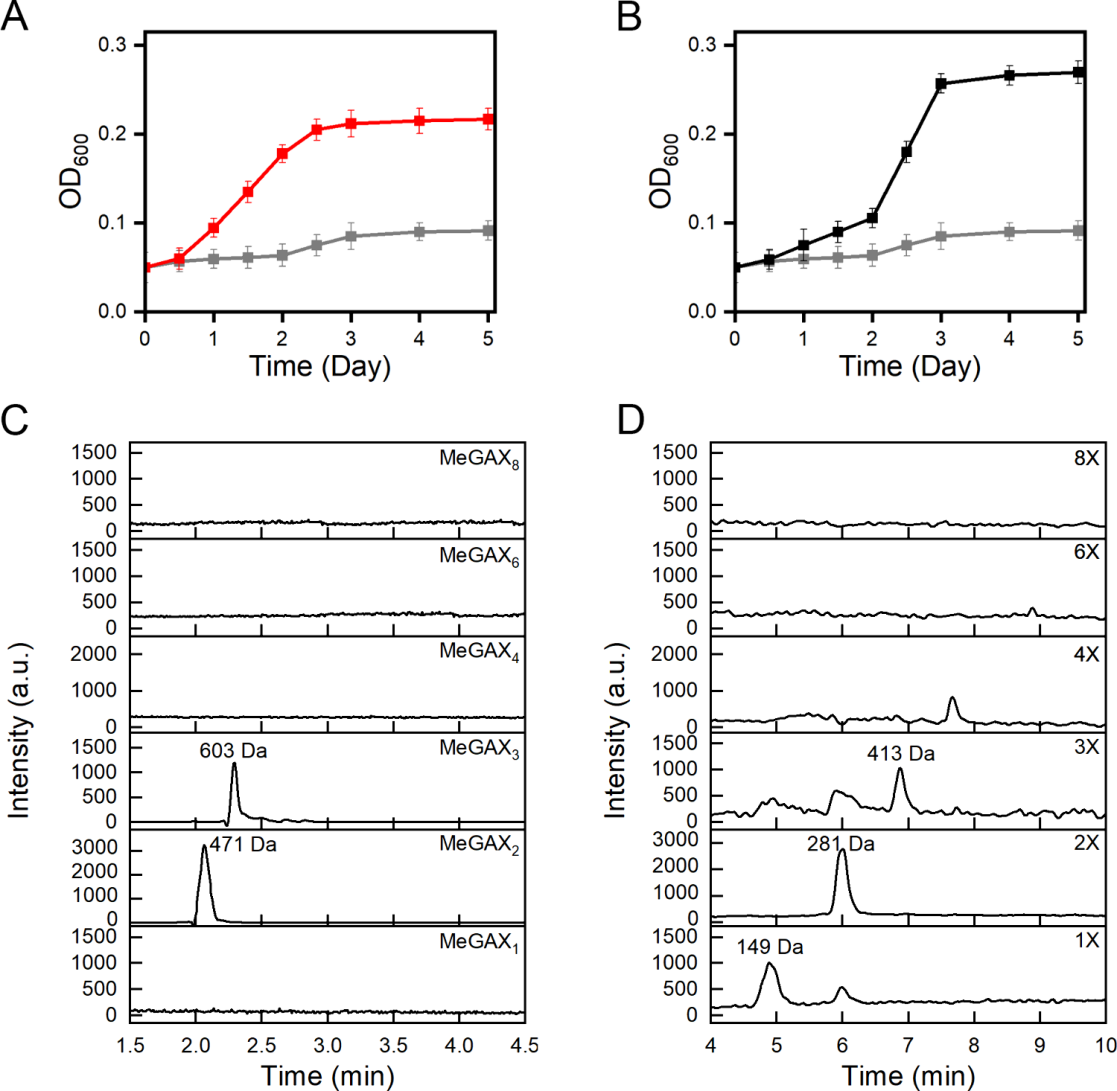
Growth of*B. altitudinis* SRL571 in glucuronoxylan-based media. Growth curves of*B. altitudinis* SRL571 in M9 minimal medium in the absence (gray line) and in the presence of xylose (A – red line) and glucuronoxylan (B – black line). The experiments were performed in triplicate, and the error bars represent SD. (C, D) HPLC-MS chromatogram of MeGAX and XOS oligosaccharides contained in the culture supernatants collected after 48 h of growth in the presence of glucuronoxylan.

A genome analysis was performed to identify potential β-xylanase and β-xylosidase enzymes in *B. altitudinis* SRL571. The analysis revealed 27 genes encoding putative glycoside hydrolases (GHs) from 20 distinct GH families. Among these, six GHs, belonging to families GH3, GH10, GH11, GH30, GH43_11, and GH43_16, are predicted to encode enzymes with β-1,4-xylosidase or β-1,4-xylanase activity (Figure S2). Notably, only the GH11, GH30, and GH43_16 enzymes (hereafter referred to as BaGH11, BaGH30, and BaGH43_16) contain signal peptides, suggesting they are likely secreted.

To further investigate the role of these secreted enzymes in xylan degradation, the xylan catabolic pathway of *B. altitudinis* SRL571 was reconstructed through comparative genomic analysis with the well-characterized xylan-degrading*B. subtilis*sp. 168,^15,16^ which shares over 95% 16S rRNA sequence identity. The comparison revealed that *B. altitudinis* SRL571 harbors all the enzymes required for xylan degradation (Figure 2 and Table S1). BaGH30 and BaGH43_16 show high sequence identity with XynC and XynD from*B. subtilis*sp. 168, respectively, while BaGH11 shares 47.9% of sequence identity with XynA. The genes encoding BaGH30 and BaGH43_16 are organized in the *xynDC* operon, mirroring the operon structure found in*B. subtilis*sp. 168.^15,16^ A key distinction between the two species lies in the genomic context of BaGH11: in *B. altitudinis* SRL571, it is located within the *xylAB* operon and regulated by the XylR repressor, whereas in *B. subtilis*sp. 168, the corresponding gene is not operon-associated.^15^ In this work, we focused on BaGH11 and BaGH30, as they are the two extracellular enzymes putatively involved in the first steps of xylan degradation.

**Figure 2:**
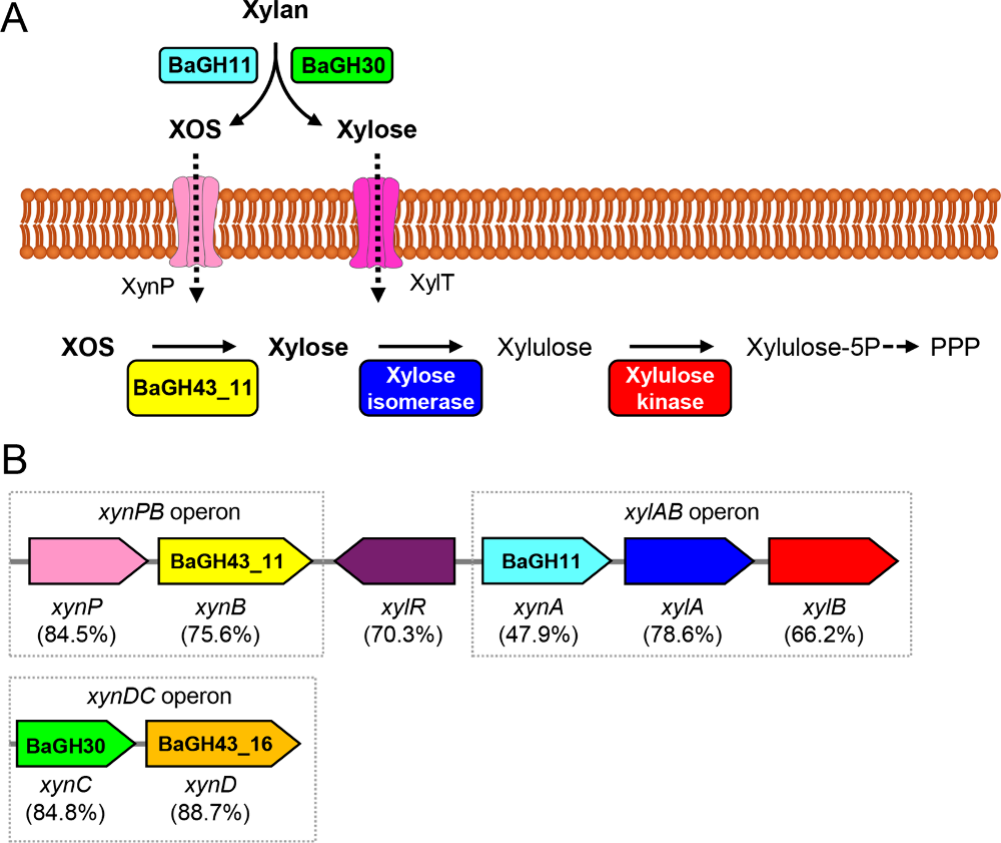
**S**chematic representation of the xylan catabolic pathway in*B. altitudinis* SRL571. (A) Xylan catabolic pathway. Extracellular BaGH11 and BaGH30 hydrolyze xylan to xylo-oligosaccharides (XOS) and xylose. XOS and xylose uptake is suggested to occur via the XylT transporter and the XynP permease, respectively. Intracellular BaGH43_11 hydrolyzes XOS to xylose, which is then converted to xylulose-5-phosphate by xylose isomerase and xylulose kinase. (B) Genomic arrangement of the enzymes and transporters involved in xylan catabolism. The color of the genes corresponds to the proteins shown in panel A. Xyl repressor (XylR in purple) controls the expression of both *xynPB* and *xylAB* operons. The names of the homologous genes in*B. subtilis*sp. 168 are reported and the amino acid sequence identity is given in parentheses.

### BaGH11 and BaGH30 Work in Synergy to Break Down Glucuronoxylan

The 3D structures of BaGH11 and BaGH30 were predicted using AlphaFold 2 ^37^ and structurally aligned with their homologues. The structural model of BaGH11 showed a typical β-jelly roll fold composed of 11 β-sheets and an α-helix, as reported in other homologous enzymes.^43^ The active cleft, consisting of the −3, −2, −1, and +1 subsites, is highly conserved in the *Bacillus* homologues (Figure 3A and Figure S3). The 3D structure of BaGH30 is organized in a catalytic domain with the (α/β)_8_ TIM barrel fold and an additional domain whose function remains unknown (Figure 3B). Structural alignment of BaGH30 with the homologues with available 3D structures indicates a highly conserved active site including the −3, −2a, −2b, −1, + 1, + 2 subsites (Figure S4). The conservation of subsites −2a and −2b suggests the ability of BaGH30 to hydrolyze glucuronoxylan, as reported for*B. subtilis*sp. 168.^44^

**Figure 3:**
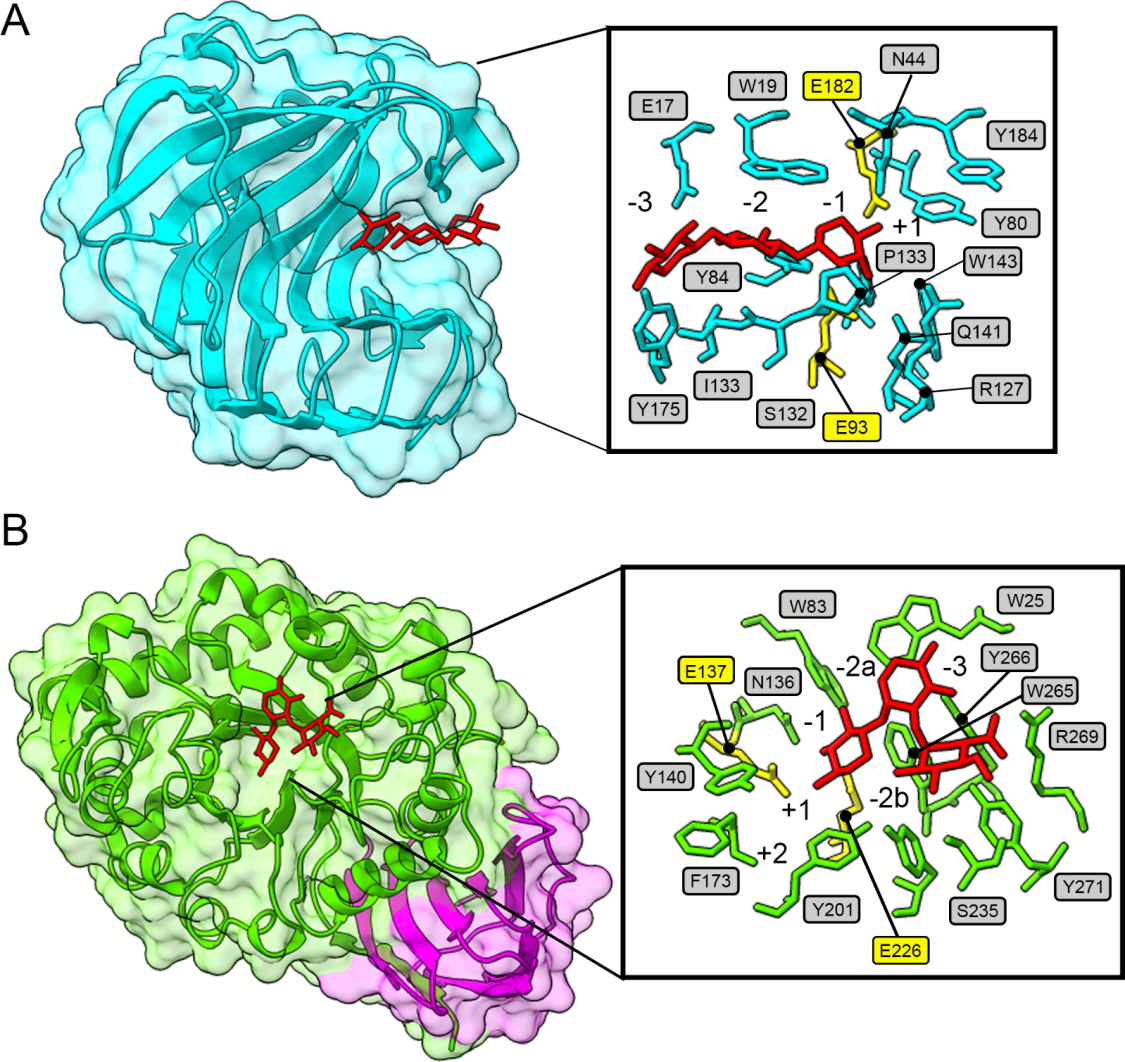
Structural analysis of BaGH11 and BaGH30. 3D model of BaGH11 (A) and BaGH30 (B) predicted with Alphafold 2.^37^ The catalytic domain is colored in cyan for BaGH11 and green for BaGH30. The additional domain of BaGH30 is shown in magenta. The active sites of BaGH11 and BaGH30 were complexed with xylotriose and 4-*O*-methyl-α-d-glucopyranuronic acid-(1–2)-β-d-xylopyranose-(1–4)-β-d-xylopyranose, respectively. The substrate molecule is represented by red sticks and was localized by structural superimposition with GH11 from *Bacillus subtilis* cocrystallized with sugar (PDB: 2QZ3, chain A) and GH30_8 from *B. subtilis* sp. 168 cocrystallized with sugar (PDB: 3KL5, chain A). The catalytic residues are represented by yellow sticks.

The hydrolytic activity of BaGH11 and BaGH30 was tested on glucuronoxylan, arabinoxylan and xyloglucan, using the DNS assay. BaGH11 is active only on glucuronoxylan and arabinoxylan yielding similar specific activities (15.2 ± 0.6 U/mg and 10.9 ± 0.4 U/mg for glucuronoxylan and arabinoxylan, respectively) with highest activity occurring at 55 °C and at pH 7.0 (Figure 4 **A and B**). The enzyme can be defined as cold-active^45,46^ as it maintains ∼ 50% of its activity at 10 °C (Figure 4A). In the case of BaGH30, the highest activity is detected at 60 °C and pH 8.0 (Figure 5 **A and B**). This enzyme also catalyzes the breakdown of glucuronoxylan and arabinoxylan, with higher specific activity with glucuronoxylan (5.4 ± 0.7 U/mg) compared to arabinoxylan (0.6 ± 0.2 U/mg).

**Figure 4:**
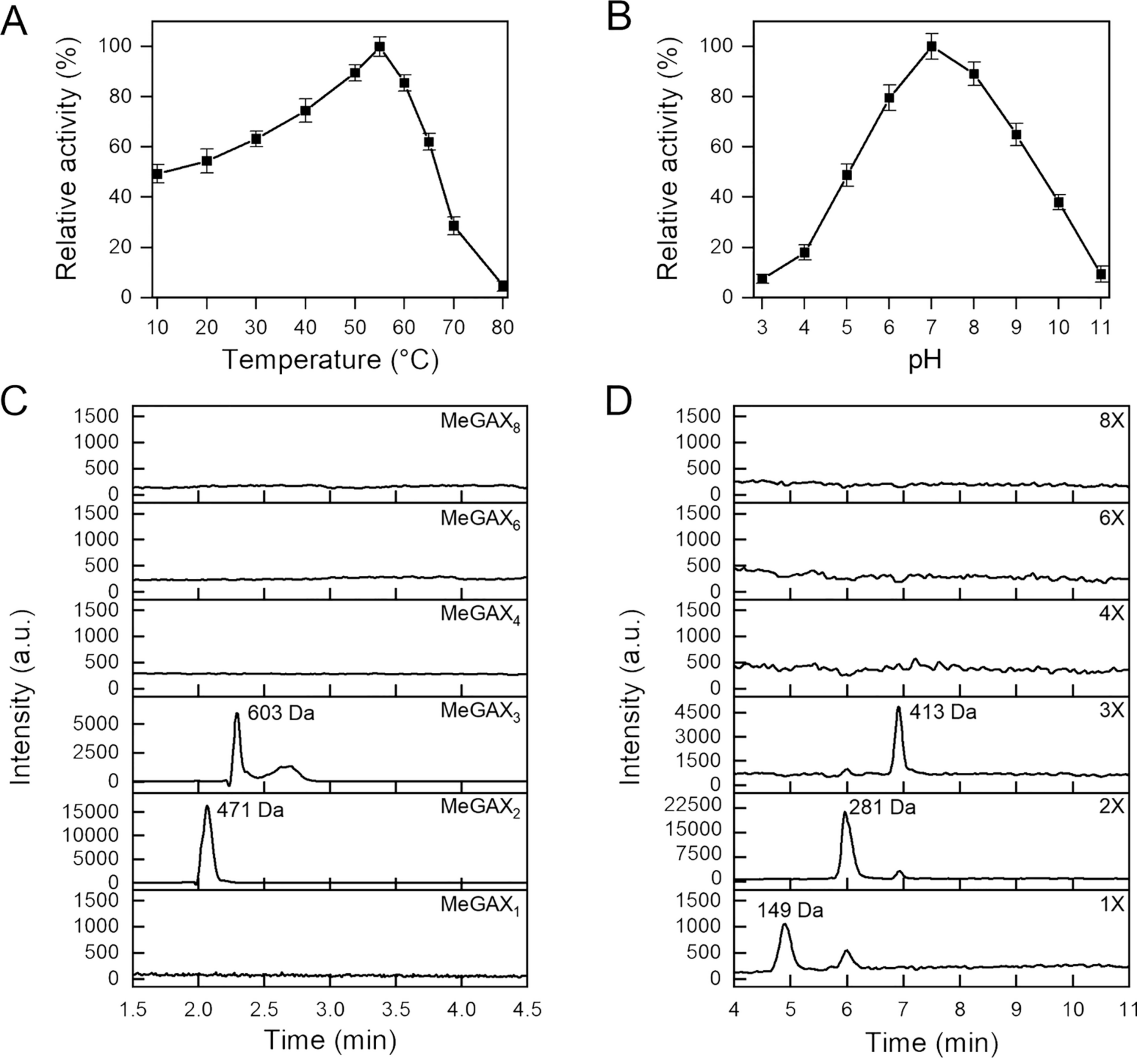
Biochemical characterization of BaGH11. Effects of temperature (A) and pH (B) on BaGH11 activity determined by the DNS assay using glucuronoxylan as a substrate. All the experiments were performed in triplicate, and the error bars refer to standard deviation (*n* = 3). HPLC-MS chromatogram of MeGAX oligosaccharides (C) and XOS (D) derived from glucuronoxylan degradation. Reactions were performed in triplicate with 10 g/L glucuronoxylan and 1 mg/mL BaGH11 at 30 °C for 24 h under shaking. One of the three HPLC-MS chromatograms is shown here.

**Figure 5:**
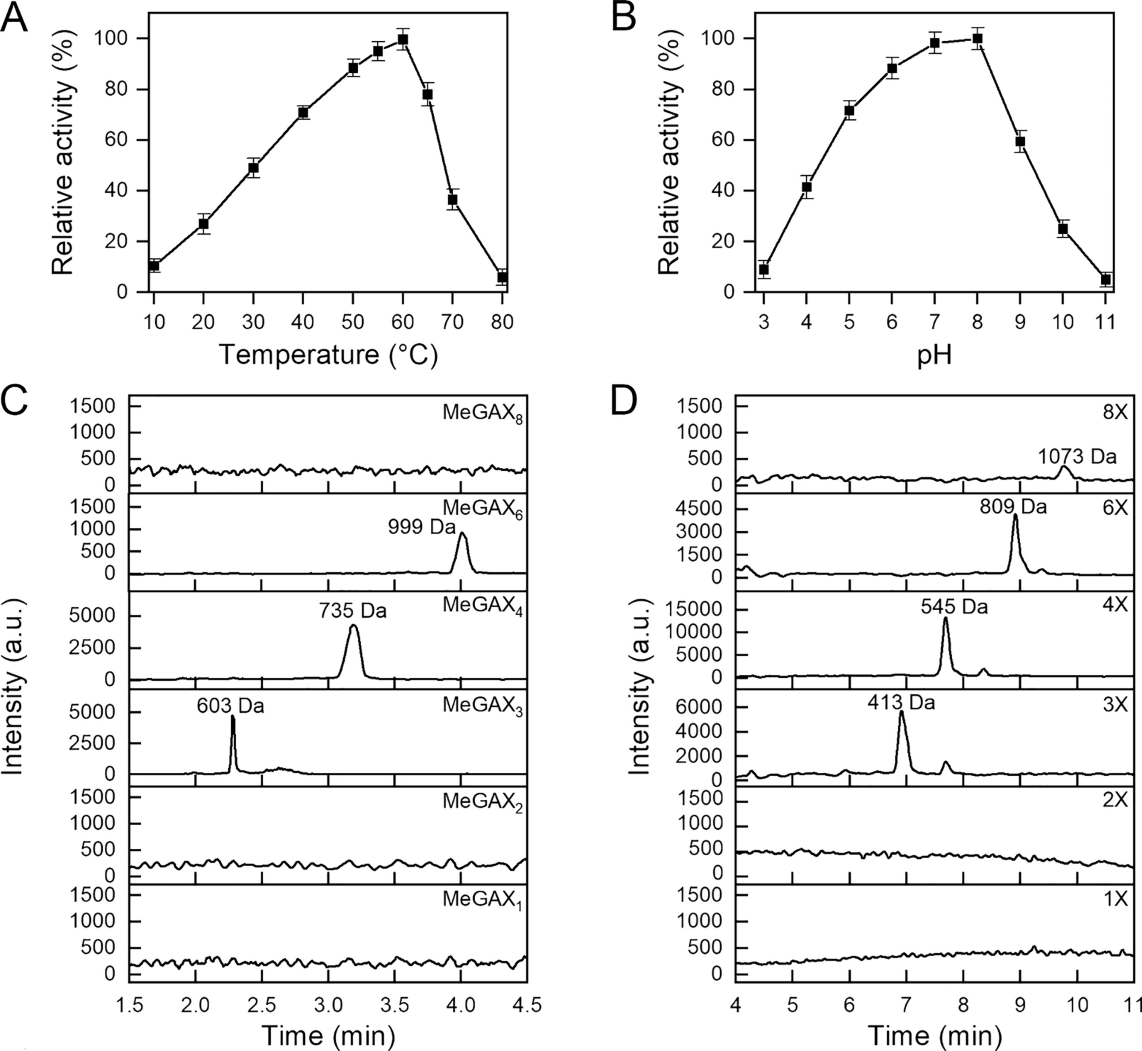
Biochemical characterization of BaGH30. Effects of temperature (A) and pH (B) on BaGH30 activity determined by the DNS assay using glucuronoxylan as a substrate. All the experiments were performed in triplicate, and the error bars refer to standard deviation (*n* = 3). HPLC-MS chromatogram of MeGAX oligosaccharides (C) and XOS (D) derived from glucuronoxylan degradation. Reactions were performed in triplicate with 10 g/L of glucuronoxylan and 1 mg/mL of BaGH30 at 30 °C for 24 h under shaking. One of the three HPLC-MS chromatograms is shown here.

HPLC-MS analysis of glucuronoxylan degradation products was performed after 24 h incubation. The chromatograms obtained after treatment with BaGH11 indicate that xylobiose (2X) and MeGAX_2_ exhibit higher intensity, while xylose (1X), xylotriose (3X), and MeGAX3 show lower intensities (Figure 4 **C and D**). Typically, GH11 enzymes exhibit an endocatalytic pattern, releasing mainly short-chain XOS.^43^ However, two GH11 enzymes (MetXyn11 and Compost21_GH11) identified by metagenomic analyses act as exoxylosidases, yielding xylobiose as their sole product.^47–49^ Sequence and structural analyses of BaGH11 indicate that it is a canonical member of the GH11 family (Figure 2 and Figure S1). Indeed, the exoactivity of MetXyn11 and Compost21_GH11 is due to the presence of two extra loops (EL1 and EL2) missing in the BaGH11 structure.^47–49^

Glucuronoxylan hydrolysis by BaGH30 releases medium molecular weight (3X to 8X) XOS and MeGAX oligosaccharides, with XOS 4X being the most intense product (Figure 5 **C and D**). Low molecular weight XOS (1X and 2X) were not detected, showing that BaGH30 is an endoxylanase active on MeGA-decorated xylan, as already suggested by structural analyses.

The combined effect of the two enzymes on glucuronoxylan degradation was assessed by a two-step experiment: *i)* glucuronoxylan was incubated with BaGH30 for 24 h and *ii)* BaGH11 was added to the reaction mixture for an additional 24 h. The degradation products were monitored by HPLC-MS after 24 and 48 h, showing that the medium-weight oligosaccharides released by BaGH30 after 24 h (Figure 6A) were completely hydrolyzed to 2X and 1X by BaGH11 after 48 h (Figure 6 **B, C**). To investigate the potential synergistic effects of these enzymes, BaGH30 and BaGH11 were combined at a 1:1 ratio. The enzymatic mixture released a quantity of reducing sugars 1.4 times higher than the sum of the individual activities of each enzyme. This finding suggests that the enzymes act synergistically in glucuronoxylan degradation (Figure 6D).

**Figure 6:**
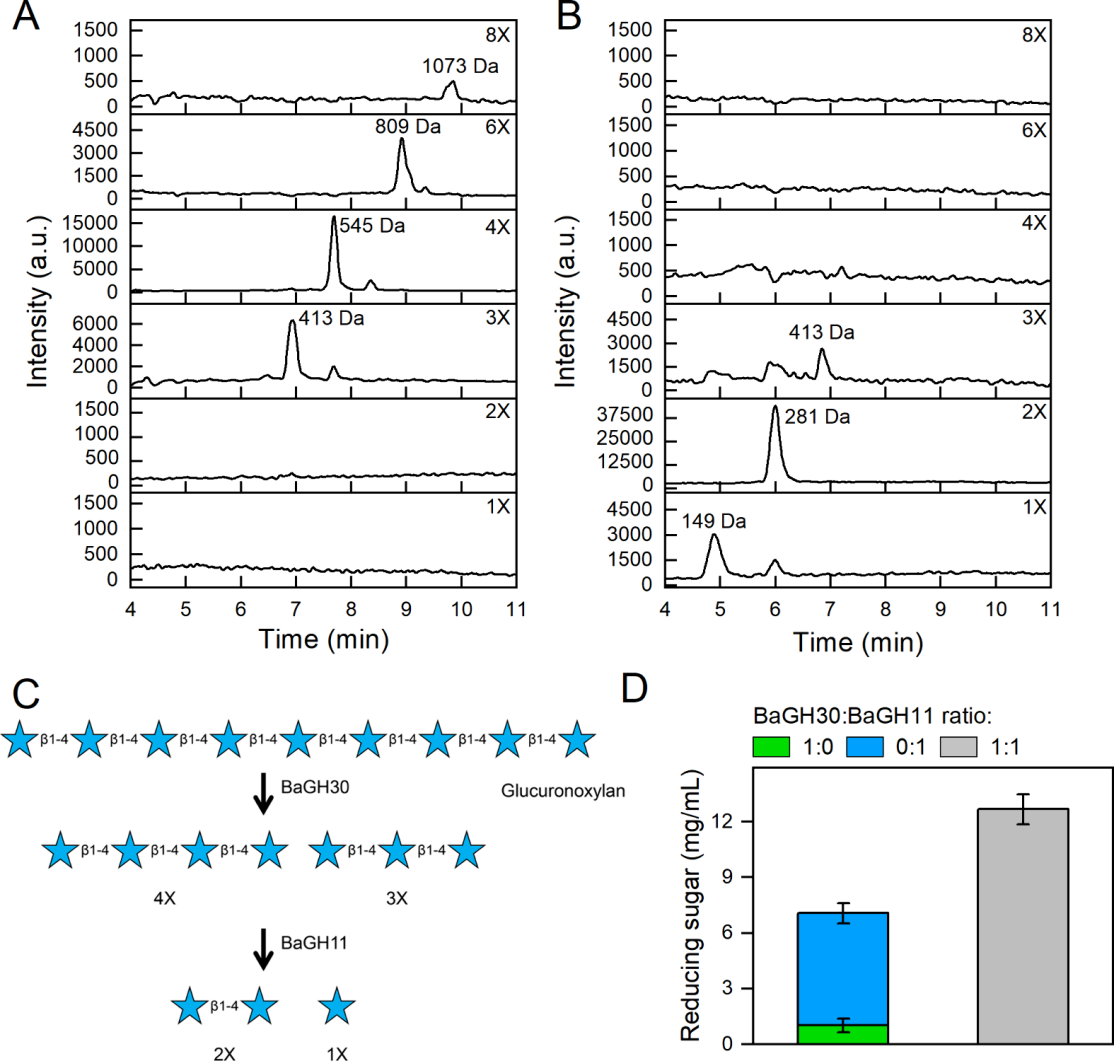
Combined effects of BaGHs on glucuronoxylan degradation. The combined effects of BaGH30 and BaGH11 were evaluated using a two-step reaction. (A) HPLC-MS chromatogram of glucuronoxylan degradation products after 24 h incubation with BaGH30 (first step). (B) HPLC-MS glucuronoxylan degradation products obtained after an additional 24 h of incubation with BaGH11 (second step). The reactions were performed in triplicate using 10 g/L glucuronoxylan at 30 °C with shaking. One of the three HPLC-MS chromatograms is shown. (C) Scheme of the glucuronoxylan degradation pathway in the presence of BaGH30 followed by treatment with BaGH11. Due to the limitations of HPLC-MS analyses, we were unable to determine the exact position of the MeGA decoration and therefore only represented the XOS. (D) Synergistic effects of BaGH30 and BaGH11. This effect was evaluated by preparing reactions in ammonium acetate buffer (pH 7.5 containing glucuronoxylan (10 g/L) and BaGH30 and BaGH11 molar ratios of 1:1, 1:0, and 0:1. The reaction mixtures were incubated for 48 h at 30 °C with shaking.

### BaGH11 and BaGH30 Are Halotolerant Enzymes

*B. altitudinis* SRL571 is a halotolerant microorganism exposed to high NaCl environmental concentrations.^30^ Therefore, we investigated the effect of salt on the two secreted enzymes. Due to the low stability of both enzymes at their *T*_*opt*_, where inactivation occurred after ∼ 15 min **(** Figure S5 **)**, the impact of salt on activity was examined at temperatures 10 °C below *T*_*opt*_ (45 °C for BaGH11 and 50 °C for BaGH30) across a wide range of NaCl concentrations. Results demonstrated that both enzymes are tolerant to NaCl concentrations up to 2.0 M. Moreover, the activity of BaGH30 increases at 1.0 and 2.0 M NaCl, surmising a possible activation effect triggered by salt (Figure 7A and **B**). Given the established role of salt in enhancing the stability of halophilic and halotolerant enzymes,^50–53^ the thermal stability of BaGH11 and BaGH30 was investigated under varying concentrations of NaCl using thermal denaturation experiments to assess structural stability and activity-based assays of kinetic stability. Thermal denaturation experiments were performed by CD spectroscopy at a fixed wavelength (220 nm) in the temperature range 10–90 °C. In the absence of NaCl, the thermostability of both enzymes was similar to unfolding transition midpoints (*T*_*m*_) of 56.0 ± 0.8 °C for BaGH11 and 59.2 ± 0.7 °C for BaGH30 (Figure 7C and **7D**). Compared to the salt-free condition, BaGH11 maintained similar *T*_*m*_ values at 1.0 and 2.0 M NaCl, with a slight decrease observed at 3.0 M NaCl (Figure 7C). In contrast, BaGH30 showed increased thermal stability at 1.0 and 2.0 M NaCl, whereas at high salt concentration the measured *T*_*m*_ was comparable to that recorded in the control without salt (Figure 7D).

**Figure 7:**
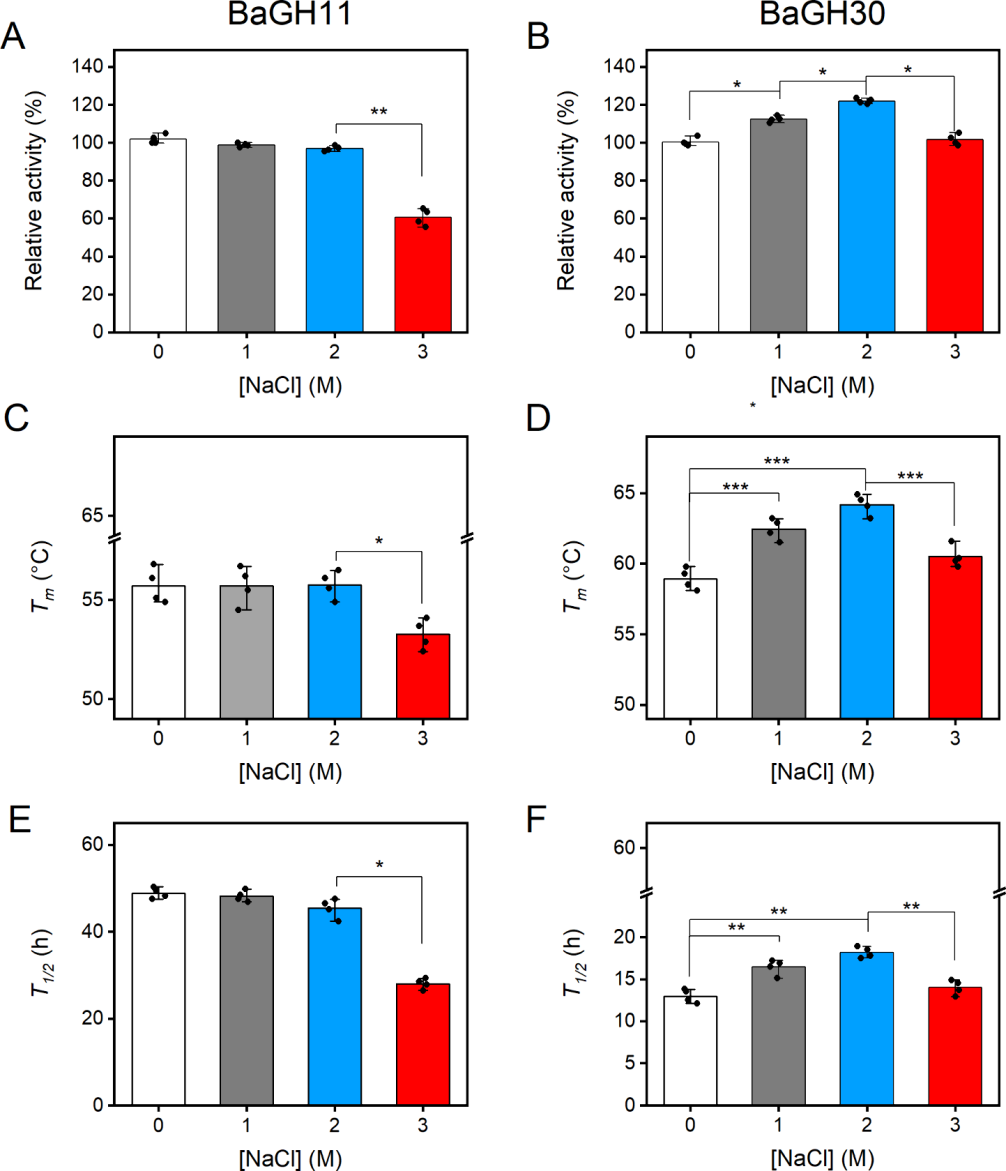
Effects of NaCl on the activity and stability of BaGH11 and BaGH30. Effects of NaCl on the activity (A, B), thermal stability (C, D), and kinetic stability (E, F) of BaGH11 and BaGH30. The relative activities of BaGH11 (A) and BaGH30 (B) were determined in the absence and presence of NaCl using glucuronoxylan as a substrate as described in material and methods section. Effects of NaCl on the *T*_m_ values of BaGH11 (C) and BaGH30 (D). *T*_m_ values were determined by monitoring the CD signal at 222 nm with increasing salt concentrations. Effects of NaCl on long-term thermal stability of BaGH11 (E) and BaGH30 (F). The long-term thermal stability was determined by incubating the enzymes in the absence or presence of NaCl at 45 °C for BaGH11 and 50 °C for BaGH30. *T*_1/2_ was calculated as described in the Materials and Methods section. Statistical analyses were performed using unpaired two-tailed Student’s *t* test, n.s.: not significant *p* > 0.05, *p < 0.05, **p < 0.01, ***p < 0.001.

The kinetic stability of BaGH11 was higher than that of BaGH30 with a half-life time (*t*_*1/2*_) in the absence of salt of 48.7 ± 0.6 h and 13.3 ± 0.7 h, respectively (Figure 7E, and **7F**). In the presence of salt, the trend was similar to that observed in thermal denaturation experiments. Indeed, BaGH11 displayed comparable *t*_*1/2*_ values in the range of 0–2.0 M NaCl but showed a significant drop at higher salt concentrations (Figure 7E). In contrast, the kinetic stability of BaGH30 increased to 1.0 and 2.0 M NaCl, followed by a decrease at 3.0 M NaCl (Figure 7F).

## Discussion

*B. altitudinis* strains, widely distributed from Arctic to Mediterranean environments, are considered a valuable source of xylanolytic enzymes.^54–56^ Among them, the halotolerant *B. altitudinis* SRL571 grows on xylan as the sole carbon source yielding short-chain XOS and MeGAX oligosaccharides as the main degradation products, differing from those of *B. altitudinis* XYL17, where XOS 5X is the dominant product.^54^ Another notable feature of *B. altitudinis* SRL571 is its ability to utilize xylose as the sole carbon source, a feature missing in *B. subtilis*sp. 168, due to the lack of a xylose-specific permease.^17,57–59^ Genomic analysis highlights that xylose uptake may be facilitated by XylT, a transporter sharing 45% amino acid sequence identity with the D-xylose-H^+^ symporter from *Lactobacillus brevis*.^60^

This study focuses on two key glycosidases involved in the initial steps of glucuronoxylan degradation. BaGH30 breaks down the glucuronoxylan backbone to medium-chain XOS and MeGAX, whereas BaGH11 further hydrolyzes glucuronoxylan and medium-chain XOS to xylose and xylobiose. Interestingly, despite containing Arg269, which interacts with the MeGA of glucuronoxylan,^61^ BaGH30 can also generate linear XOS. The similarity between the glucuronoxylan degradation products observed *in vitro* and those detected in the supernatants of cell cultures, along with the presence of a signal peptide for secretion in the coding sequence, suggest that BaGH11 and BaGH30 act together in the extracellular environment. The synergistic action of these enzymes enhances the release of XOS and xylose from glucuronoxylan polysaccharide, consistent with previously reported activities of GH30 and GH11 enzymes,^15,62^ such as XynA and XynC from *B. subtilis*sp. 168.

BaGH30 is homologous to XynC a β-xylanase from*B. subtilis*sp. 168, and its encoding gene is a part of the *xynDC* operon. In*B. subtilis*sp. 168, this operon is constitutively expressed and plays a key role in glucuronoxylan and arabinoxylan degradation.^15,61,63^ By contrast, the gene encoding BaGH11 is located within an operon under the control of the XylR repressor. This regulatory arrangement distinguishes *B. altitudinis* SRL571 from*B. subtilis*sp. 168, where the orthologous *xynA* is not part of any operon and is constitutively expressed.^15^ The glucuronoxylan degradation products generated by the action of BaGH30 and BaGH11 can be uptake into *B. altitudinis* SRL571 cells via the sugar transporters XylT and XynP. It is noteworthy that XynP shares 84.5% amino acid sequence identity with its homologue from*B. subtilis*sp. 168. However, further investigation of sugar transport, particularly the role of XylT, will be critical to understanding xylose metabolism in *B. altitudinis* SRL571.

Consistent with their origin, both enzymes under study tolerate NaCl concentrations up to 2.0 M but exhibit distinct behaviors. BaGH11 is tolerant to high salt concentrations, while BaGH30 shows salt-induced stabilization at 1.0 and 2.0 M NaCl. Our experimental data suggests an improvement in BaGH30 stability at these salt concentrations, resulting in a higher amount of released sugar over the incubation period. Although we cannot entirely rule out salt-activation mechanisms for BaGH30 at 1.0 and 2.0 M NaCl, further investigation is needed to address technical issues related to the DNS assay observed in Michaelis–Menten experiments, particularly at low substrate concentrations. While there is no clear definition of salt-tolerant xylanases, both BaGH30 and BaGH11 can be classified as such, given their ability to maintain activity and stability at salt concentrations exceeding 0.6 M, which is the average salinity of oceans.^9^

Two salt-adaptation mechanisms have been reported for xylanases: (i) abundance of acidic amino acids,^64,65^ and (ii) high structural flexibility to avoid structural collapse triggered by high salt concentrations.^66^ Structural analyses of BaGH11 and its homologues from both halophilic and nonhalophilic bacteria did not reveal any clear correlation between their origin and their overall surface charge (Figure S6). This finding, combined with the observation that BaGH11 is also cold-active (it retains 50% activity at 10 °C),^67,68^ suggests that its halotolerance is likely to rely on high structural flexibility. Coupling of cold activity and halotolerance has been observed in several xylanases belonging to GH10 and GH11 families isolated from marine and mesophilic bacteria.^69–71^

For BaGH30, the salt adaptation appears to follow a different mechanism. While an abundance of surface acidic residues is a known halotolerance strategy,^64,65^ a direct comparison between BaGH30 and its nonhalotolerant homologous from *Clostridium thermocellum* did not reveal significant differences in the overall surface electrostatic potential (Figure S7). The paucity of studies on salt tolerance in other GH30_8 members hinders the identification of salt-adaptation mechanisms. Moreover, we cannot completely exclude the possibility that GH30_8s, being secreted enzymes, have evolved salt tolerance mechanisms to counteract fluctuations in salt concentrations typical of extracellular environments.

In conclusion, this study elucidates the remarkable capability of *B. altitudinis* SRL571 to degrade glucuronoxylan through two salt-resistant xylanases belonging to the GH families 11 and 30_8. The synergistic effect of BaGH30 and BaGH11 in the efficient degradation of glucuronoxylan to xylose and xylobiose highlights the importance of using enzymes with complementary activities and substrate specificities for effective biomass degradation in saline environments.

## Supplementary Material



## Notes

List of the homologous genes involved in xylan degradation in *Bacillus subtilis*sp. 168 (Table S1); glucuronoxylan degradation by halo-assay (Figure S1); annotation of the *B. altitudinis* SRL571 GHs and the glucuronoxylan degradation pathway in *B. subtilis*sp. 168 (Figure S2); structural alignment of BaGH11 and its homologous (Figure S3) and of BaGH30 and its homologous (Figure S4); thermal stability of BaGHs at *T*_opt_ (Figure S5); surface charge analysis of BaGH11 and BaGH30 (Figures S6 and S7, respectively) (PDF)

## Abbreviations

2X: xylobiose
3X: xylotriose
4X: xylotetraose
6X: xylohexaose
8X: xylooctaose
CD: circular dichroism
DNS: dinitrosalicylic acid
GH: glycoside hydrolase
BaGH11: glycoside hydrolase belonging to family 11 identified in the genome of *Bacillus altitudinis* strain SRL571
HPLC-MS: high-performance liquid chromatography–mass spectrometry
MeGAX: methyl glucuronoxylan
PB: phosphate buffer
Tm: unfolding transition midpoint temperature
Topt: optimum temperature of the catalysis
t1/2: enzyme half-life time
XOS: xylo-oligosaccharides.

## References

[1] Prampolini M., Savini A., Foglini F., Soldati M. (2020). Seven Good Reasons
for Integrating Terrestrial and Marine Spatial Datasets in Changing
Environments. Water.

[2] Dutschei T., Beidler I., Bartosik D., Seeßelberg J., Teune M., Bäumgen M., Ferreira S. Q., Heldmann J., Nagel F., Krull J., Berndt L., Methling K., Hein M., Becher D., Langer P., Delcea M., Lalk M., Lammers M., Höhne M., Hehemann J., Schweder T., Bornscheuer U. T. (2023). Marine *Bacteroidetes* Enzymatically Digest Xylans from Terrestrial
Plants. Environmental Microbiology.

[3] Herrmann N., Boom A., Carr A. S., Chase B. M., Granger R., Hahn A., Zabel M., Schefuß E. (2016). Sources, Transport
and Deposition of Terrestrial Organic Material: A Case Study from
Southwestern Africa. Quaternary Science Reviews.

[4] Chen Z., Li S., Fu Y., Li C., Chen D., Chen H. (2019). Arabinoxylan
Structural Characteristics, Interaction with Gut Microbiota and Potential
Health Functions. Journal of Functional Foods.

[5] Bastawde K. B. (1992). Xylan Structure,
Microbial Xylanases, and Their Mode of Action. World J. Microbiol. Biotechnol..

[6] Kormelink F. J. M., Voragen A. G. J. (1993). Degradation of
Different [(Glucurono)­Arabino]­Xylans
by a Combination of Purified Xylan-Degrading Enzymes. Appl. Microbiol. Biotechnol..

[7] Collins T., Gerday C., Feller G. (2005). Xylanases, Xylanase Families and
Extremophilic Xylanases. FEMS Microbiol Rev..

[8] Qeshmi F. I., Homaei A., Fernandes P., Hemmati R., Dijkstra B. W., Khajeh K. (2020). Xylanases from Marine Microorganisms: A Brief Overview
on Scope, Sources, Features and Potential Applications. Biochimica et Biophysica Acta (BBA) - Proteins and Proteomics.

[9] Cao L., Zhang R., Zhou J., Huang Z. (2021). Biotechnological Aspects
of Salt-Tolerant Xylanases: A Review. J. Agric.
Food Chem..

[10] Nguyen S. T. C., Freund H. L., Kasanjian J., Berlemont R. (2018). Function,
Distribution, and Annotation of Characterized Cellulases, Xylanases,
and Chitinases from CAZy. Appl. Microbiol. Biotechnol..

[11] Juturu V., Wu J. C. (2012). Microbial Xylanases: Engineering, Production and Industrial Applications. Biotechnology Advances.

[12] Drula E., Garron M.-L., Dogan S., Lombard V., Henrissat B., Terrapon N. (2022). The Carbohydrate-Active
Enzyme Database: Functions
and Literature. Nucleic Acids Res..

[13] Ahmed S., Riaz S., Jamil A. (2009). Molecular Cloning of Fungal Xylanases:
An Overview. Appl. Microbiol. Biotechnol..

[14] Beg Q. K., Kapoor M., Mahajan L., Hoondal G. S. (2001). Microbial Xylanases
and Their Industrial Applications: A Review. Appl. Microbiol. Biotechnol..

[15] Rhee M. S., Wei L., Sawhney N., Kim Y. S., Rice J. D., Preston J. F. (2016). Metabolic
Potential of Bacillus Subtilis 168 for the Direct Conversion of Xylans
to Fermentation Products. Appl. Microbiol. Biotechnol..

[16] Rhee M. S., Wei L., Sawhney N., Rice J. D., St. John F. J., Hurlbert J. C., Preston J. F. (2014). Engineering the Xylan Utilization System in Bacillus
Subtilis for Production of Acidic Xylooligosaccharides. Appl. Environ. Microbiol..

[17] Singh K. D., Schmalisch M. H., Stülke J., Görke B. (2008). Carbon Catabolite
Repression in Bacillus Subtilis: Quantitative Analysis of Repression
Exerted by Different Carbon Sources. J. Bacteriol..

[18] Jordan D. B., Wagschal K., Grigorescu A. A., Braker J. D. (2013). Highly Active β-Xylosidases
of Glycoside Hydrolase Family 43 Operating on Natural and Artificial
Substrates. Appl. Microbiol. Biotechnol..

[19] Xiao F., Zhang Y., Zhang L., Li S., Chen W., Shi G., Li Y. (2024). Advancing Bacillus Licheniformis as a Superior Expression
Platform through Promoter Engineering. Microorganisms.

[20] Li Y., Liu X., Zhang L., Ding Z., Xu S., Gu Z., Shi G. (2019). Transcriptional Changes in the Xylose Operon in Bacillus Licheniformis
and Their Use in Fermentation Optimization. IJMS.

[21] Bazos, I. ; Kokkoris, I. P. ; Dimopoulos, P. Diversity of Halophytes and Salt Tolerant Plants at the Species-, Habitats- and High-Rank Syntaxa Level in Greece. In Handbook of Halophytes; Grigore, M.-N. , Ed.; Springer International Publishing: Cham, 2021; pp 787–820. 10.1007/978-3-030-57635-6_26.

[22] Tilman D., Lehman C. L., Thomson K. T. (1997). Plant Diversity
and Ecosystem Productivity:
Theoretical Considerations. Proc. Natl. Acad.
Sci. U.S.A..

[23] Reang L., Bhatt S., Tomar R. S., Joshi K., Padhiyar S., Vyas U. M., Kheni J. K. (2022). Plant Growth Promoting Characteristics
of Halophilic and Halotolerant Bacteria Isolated from Coastal Regions
of Saurashtra Gujarat. Sci. Rep.

[24] Barajas
González J. A., de la Rosa Y. E. K., Carrillo-González R., González-Chávez M. D. C. Á., Hidalgo Lara M. E., Soto Hernández R. M., Herrera Cabrera B. E. (2024). NaCl Modifies
Biochemical Traits in Bacterial Endophytes Isolated from Halophytes:
Towards Salinity Stress Mitigation Using Consortia. Plants.

[25] Szymańska S., Płociniczak T., Piotrowska-Seget Z., Złoch M., Ruppel S., Hrynkiewicz K. (2016). Metabolic
Potential and Community
Structure of Endophytic and Rhizosphere Bacteria Associated with the
Roots of the Halophyte Aster Tripolium L. Microbiological
Research.

[26] Khan, A. L. ; Shahzad, R. ; Al-Harrasi, A. ; Lee, I.-J. Endophytic Microbes: A Resource for Producing Extracellular Enzymes. In Endophytes: Crop Productivity and Protection; Maheshwari, D. K. , Annapurna, K. , Eds.; Sustainable Development and Biodiversity; Springer International Publishing: Cham, 2017; Vol. 16, pp 95–110. 10.1007/978-3-319-66544-3_5.

[27] Ryan R. P., Germaine K., Franks A., Ryan D. J., Dowling D. N. (2008). Bacterial
Endophytes: Recent Developments and Applications. FEMS Microbiology Letters.

[28] Schulz B., Boyle C., Draeger S., Römmert A.-K., Krohn K. (2002). Endophytic Fungi: A Source of Novel
Biologically Active Secondary
Metabolites. Mycological Research.

[29] DasSarma S., DasSarma P. (2015). Halophiles and Their Enzymes: Negativity Put to Good
Use. Curr. Opin. Microbiol..

[30] Christakis C. A., Daskalogiannis G., Chatzaki A., Markakis E. A., Mermigka G., Sagia A., Rizzo G. F., Catara V., Lagkouvardos I., Studholme D. J., Sarris P. F. (2021). Endophytic Bacterial Isolates From
Halophytes Demonstrate Phytopathogen Biocontrol and Plant Growth Promotion
Under High Salinity. Front. Microbiol..

[31] Teather R. M., Wood P. J. (1982). Use of Congo Red-Polysaccharide
Interactions in Enumeration
and Characterization of Cellulolytic Bacteria from the Bovine Rumen. Appl. Environ. Microbiol..

[32] Wistrand M., Sonnhammer E. L. (2005). Improved
Profile HMM Performance by Assessment of Critical
Algorithmic Features in SAM and HMMER. BMC Bioinformatics.

[33] Orlando M., Marchetti A., Bombardi L., Lotti M., Fusco S., Mangiagalli M. (2025). Polysaccharide
Degradation in an Antarctic Bacterium:
Discovery of Glycoside Hydrolases from Remote Regions of the Sequence
Space. Int. J. Biol. Macromol..

[34] Mitchell A. L., Attwood T. K., Babbitt P. C., Blum M., Bork P., Bridge A., Brown S. D., Chang H.-Y., El-Gebali S., Fraser M. I., Gough J., Haft D. R., Huang H., Letunic I., Lopez R., Luciani A., Madeira F., Marchler-Bauer A., Mi H., Natale D. A., Necci M., Nuka G., Orengo C., Pandurangan A. P., Paysan-Lafosse T., Pesseat S., Potter S. C., Qureshi M. A., Rawlings N. D., Redaschi N., Richardson L. J., Rivoire C., Salazar G. A., Sangrador-Vegas A., Sigrist C. J. A., Sillitoe I., Sutton G. G., Thanki N., Thomas P. D., Tosatto S. C. E., Yong S.-Y., Finn R. D. (2019). InterPro
in 2019: Improving Coverage, Classification and Access to Protein
Sequence Annotations. Nucleic Acids Res..

[35] Teufel F., Almagro Armenteros J. J., Johansen A. R., Gíslason M. H., Pihl S. I., Tsirigos K. D., Winther O., Brunak S., von Heijne G., Nielsen H. (2022). SignalP 6.0 Predicts All Five Types
of Signal Peptides Using Protein Language Models. Nat. Biotechnol..

[36] Taboada B., Estrada K., Ciria R., Merino E. (2018). Operon-Mapper: A Web
Server for Precise Operon Identification in Bacterial and Archaeal
Genomes. Bioinformatics.

[37] Jumper J., Evans R., Pritzel A., Green T., Figurnov M., Ronneberger O., Tunyasuvunakool K., Bates R., Žídek A., Potapenko A., Bridgland A., Meyer C., Kohl S. A. A., Ballard A. J., Cowie A., Romera-Paredes B., Nikolov S., Jain R., Adler J., Back T., Petersen S., Reiman D., Clancy E., Zielinski M., Steinegger M., Pacholska M., Berghammer T., Bodenstein S., Silver D., Vinyals O., Senior A. W., Kavukcuoglu K., Kohli P., Hassabis D. (2021). Highly Accurate Protein
Structure Prediction with AlphaFold. Nature.

[38] Holm L., Laiho A., Törönen P., Salgado M. (2023). DALI Shines
a Light on Remote Homologs: One Hundred Discoveries. Protein Sci..

[39] Sievers F., Wilm A., Dineen D., Gibson T. J., Karplus K., Li W., Lopez R., McWilliam H., Remmert M., Söding J., Thompson J. D., Higgins D. G. (2011). Fast, Scalable Generation of High-quality
Protein Multiple Sequence Alignments Using Clustal Omega. Mol. Syst. Biol..

[40] Studier F. W. (2005). Protein
Production by Auto-Induction in High-Density Shaking Cultures. Protein Expression Purif..

[41] Marchetti A., Orlando M., Bombardi L., Fusco S., Mangiagalli M., Lotti M. (2024). Evolutionary History
and Activity towards Oligosaccharides and Polysaccharides
of GH3 Glycosidases from an Antarctic Marine Bacterium. Int. J. Biol. Macromol..

[42] Miller G. L. (1959). Use of
Dinitrosalicylic Acid Reagent for Determination of Reducing Sugar. Anal. Chem..

[43] Paës G., Berrin J.-G., Beaugrand J. (2012). GH11 Xylanases: Structure/Function/Properties
Relationships and Applications. Biotechnology
Advances.

[44] St
John F. J., Hurlbert J. C., Rice J. D., Preston J. F., Pozharski E. (2011). Ligand Bound Structures of a Glycosyl Hydrolase Family
30 Glucuronoxylan Xylanohydrolase. J. Mol. Biol..

[45] Collins T., Feller G. (2023). Psychrophilic Enzymes: Strategies for Cold-Adaptation. Essays in Biochemistry.

[46] Mangiagalli M., Brocca S., Orlando M., Lotti M. (2020). The “Cold Revolution”.
Present and Future Applications of Cold-Active Enzymes and Ice-Binding
Proteins. New Biotechnology.

[47] Mello B. L., Alessi A. M., Riaño-Pachón D. M., deAzevedo E. R., Guimarães F. E.
G., Espirito
Santo M. C., McQueen-Mason S., Bruce N. C., Polikarpov I. (2017). Targeted Metatranscriptomics
of Compost-Derived Consortia Reveals a GH11 Exerting an Unusual Exo-1,4-β-Xylanase
Activity. Biotechnol Biofuels.

[48] Evangelista D. E., de Oliveira Arnoldi Pellegrini V., Santo M. E., McQueen-Mason S., Bruce N. C., Polikarpov I. (2019). Biochemical
Characterization and
Low-Resolution SAXS Shape of a Novel GH11 Exo-1,4-β-Xylanase
Identified in a Microbial Consortium. Appl.
Microbiol. Biotechnol..

[49] Kadowaki M. A. S., Briganti L., Evangelista D. E., Echevarría-Poza A., Tryfona T., Pellegrini V. O. A., Nakayama D. G., Dupree P., Polikarpov I. (2021). Unlocking
the Structural Features for the Xylobiohydrolase
Activity of an Unusual GH11 Member Identified in a Compost-derived
Consortium. Biotech & Bioengineering.

[50] Ortega G., Laín A., Tadeo X., López-Méndez B., Castaño D., Millet O. (2011). Halophilic Enzyme Activation Induced
by Salts. Sci. Rep.

[51] Cen Y.-K., Zhang L., Liu M.-P., Xiang C., Lu T.-X., Xue Y.-P., Zheng Y.-G. (2025). Salt-Driven
Dynamic Folding of Halophile-Origin
Enzymes: Insights into Evolution and Protein Exploitation. Int. J. Biol. Macromol..

[52] Miyashita Y., Ohmae E., Nakasone K., Katayanagi K. (2015). Effects of
Salt on the Structure, Stability, and Function of a Halophilic Dihydrofolate
Reductase from a Hyperhalophilic Archaeon, Haloarcula Japonica Strain
TR-1. Extremophiles.

[53] Sinha R., Khare S. K. (2014). Protective Role
of Salt in Catalysis and Maintaining
Structure of Halophilic Proteins against Denaturation. Front. Microbiol..

[54] Phukon L. C., Abedin M. M., Chourasia R., Singh S. P., Tayung K., Rai A. K. (2024). Valorization of Agro-Wastes by Bacillus Altitudinis
XYL17 through Simultaneous Production of Xylanase, Xylooligosaccharides,
and Antioxidant Compounds. Industrial Crops
and Products.

[55] Ketsakhon P., Thammasittirong A., Thammasittirong S. N.-R. (2023). Adding Value to Rice Straw Waste
for High-Level Xylanase Production Using a New Isolate of Bacillus
Altitudinis RS3025. Folia Microbiol.

[56] Adhyaru D. N., Bhatt N. S., Modi H. A. (2014). Enhanced
Production of Cellulase-Free,
Thermo-Alkali-Solvent-Stable Xylanase from Bacillus Altitudinis DHN8,
Its Characterization and Application in Sorghum Straw Saccharification. Biocatalysis and Agricultural Biotechnology.

[57] Schmiedel D., Hillen W. (1996). A *Bacillus
Subtilis* 168 Mutant with
Increased Xylose Uptake Can Utilize Xylose as Sole Carbon Source. FEMS Microbiology Letters.

[58] Schmiedel D., Hillen W. (1996). Contributions of XylR,
CcpA and Cre to Diauxic Growth
of Bacillus Megaterium and to Xylose Isomerase Expression in the Presence
of Glucose and Xylose. Mol. Gen Genet.

[59] Lindner C., Stulke J., Hecker M. (1994). Regulation
of Xylanolytic Enzymes
in Bacillus Subtilis. Microbiology.

[60] Chaillou S., Bor Y.-C., Batt C. A., Postma P. W., Pouwels P. H. (1998). Molecular
Cloning and Functional Expression in Lactobacillus Plantarum 80 of
XylT, Encoding the d -Xylose–H+ Symporter of Lactobacillus
Brevis. Appl. Environ. Microbiol..

[61] St.
John F. J., Rice J. D., Preston J. F. (2006). Characterization
of XynC from *Bacillus Subtilis* Subsp. *Subtilis* Strain 168 and Analysis of Its Role in Depolymerization of Glucuronoxylan. J. Bacteriol..

[62] Vacilotto M. M., de Araujo Montalvão L., Pellegrini V. de O. A., Liberato M. V., de Araujo E. A., Polikarpov I. (2024). Two-Domain
GH30 Xylanase from Human Gut Microbiota as a Tool for Enzymatic Production
of Xylooligosaccharides: Crystallographic Structure and a Synergy
with GH11 Xylosidase. Carbohydr. Polym..

[63] Bourgois T. M., Van Craeyveld V., Van Campenhout S., Courtin C. M., Delcour J. A., Robben J., Volckaert G. (2007). Recombinant Expression and Characterization
of XynD from Bacillus Subtilis Subsp. Subtilis ATCC 6051: A GH 43
Arabinoxylan Arabinofuranohydrolase. Appl. Microbiol.
Biotechnol..

[64] Huang X., Lin J., Ye X., Wang G. (2015). Molecular
Characterization of a Thermophilic
and Salt- and Alkaline-Tolerant Xylanase from Planococcus Sp. SL4,
a Strain Isolated from the Sediment of a Soda Lake. Journal of Microbiology and Biotechnology.

[65] Xu B., Dai L., Li J., Deng M., Miao H., Zhou J., Mu Y., Wu Q., Tang X., Yang Y., Ding J., Han N., Huang Z. (2016). Molecular and Biochemical Characterization of a Novel
Xylanase from Massilia Sp. RBM26 Isolated from the Feces of Rhinopithecus
Bieti. Journal of Microbiology and Biotechnology.

[66] Zhang R., Li N., Liu Y., Han X., Tu T., Shen J., Xu S., Wu Q., Zhou J., Huang Z. (2019). Biochemical and Structural
Properties of a Low-Temperature-Active Glycoside Hydrolase Family
43 β-Xylosidase: Activity and Instability at High Neutral Salt
Concentrations. Food Chem..

[67] Feller G. (2010). Protein Stability
and Enzyme Activity at Extreme Biological Temperatures. J. Phys.: Condens. Matter.

[68] Feller G., Gerday C. (2003). Psychrophilic Enzymes:
Hot Topics in Cold Adaptation. Nat. Rev. Microbiol.

[69] Guo B., Li P.-Y., Yue Y.-S., Zhao H.-L., Dong S., Song X.-Y., Sun C.-Y., Zhang W.-X., Chen X.-L., Zhang X.-Y., Zhou B.-C., Zhang Y.-Z. (2013). Gene Cloning, Expression
and Characterization of a Novel Xylanase from the Marine Bacterium,
Glaciecola Mesophila KMM241. Marine Drugs.

[70] Wang C.-Y., Chan H., Lin H.-T., Shyu Y.-T. (2010). Production, Purification
and Characterisation of a Novel Halostable Xylanase from Bacillus
Sp. NTU-06. Annals of Applied Biology.

[71] Han Z., Shang-guan F., Yang J. (2018). Characterization of a Novel Cold-Active
Xylanase from Luteimonas Species. World J. Microbiol.
Biotechnol..

