# Supplementary material for: Xylan Degradation in the Halotolerant Bacterium Bacillus altitudinis relies on glycosidic hydrolases from families 11 and 30
Source: J Agric Food Chem. 2025 Oct 16;73(43):27599–610. doi: 10.1021/acs.jafc.5c06247 (PMC12576815; doi:10.1021/acs.jafc.5c06247)
Supplement: Supplementary file 1 [file jf5c06247_si_001.pdf]

Supplementary materials for

Xylan degradation in the halotolerant bacterium *Bacillus altitudinis* relies on glycosidic hydrolases from families 11 and 30

Alessandro Marchetti[1], Marco Orlando[1], Stefania Digiovanni[1], Christos Christakis[3], Vasileios Tsopanakis[4], Nikolaos Arapitsas[2], Ioannis V. Pavlidis[4], Panagiotis Sarris[2,3,5]*, Marco Mangiagalli[1]*, Marina Lotti[1]

[1]Department of Biotechnology and Biosciences, University of Milano-Bicocca, Piazza della Scienza 2, 20126 Milano, Italy

[2]Department of Biology, University of Crete, Voutes University Campus, 71409 Heraklion. Greece [3]IMBB FORTH, Nik. Plastira Str. Vasilika Vouton, 70013 Heraklion, Greece

[4]Department of Chemistry, University of Crete, Voutes University Campus, 71409 Heraklion. Greece

[5]University of Exeter, School of Life Sciences, Stocker Road EX4 4QD Exeter, United Kingdom

Table S1. List of homolog genes involved in xylan and xylose catabolism from *B. subtilis* sp. 168.

Gene	Function	Species	UNIPROT	Aminoacid identity (%)
xylA	xylose isomerase	*B. subtilis* sp. 168	P0CI80	78.6
xylB	xylulose kinase	*B. subtilis* sp. 168	P39211	66.2
xylR	xylose repressor	*B. subtilis* sp. 168	P94490	70.3
xylT	sugar transport	*Lactobacillus brevis*	O52733	45
xynP	sugar transport	*B. subtilis* sp. 168	P94488	84.5
xynA	extracellular β-xylanases (GH11)	*B. subtilis* sp. 168	P18429	47.9
xynB	intracellular β-xylanases (GH43_11)	*B. subtilis* sp. 168	P94489	75.6
xynC	extracellular β-xylanases (GH30_8)	*B. subtilis* sp. 168	Q45070	84.8
xynD	α-L-arabinofuranosidase (GH43_16)	*B. subtilis* sp. 168	Q45071	88.7

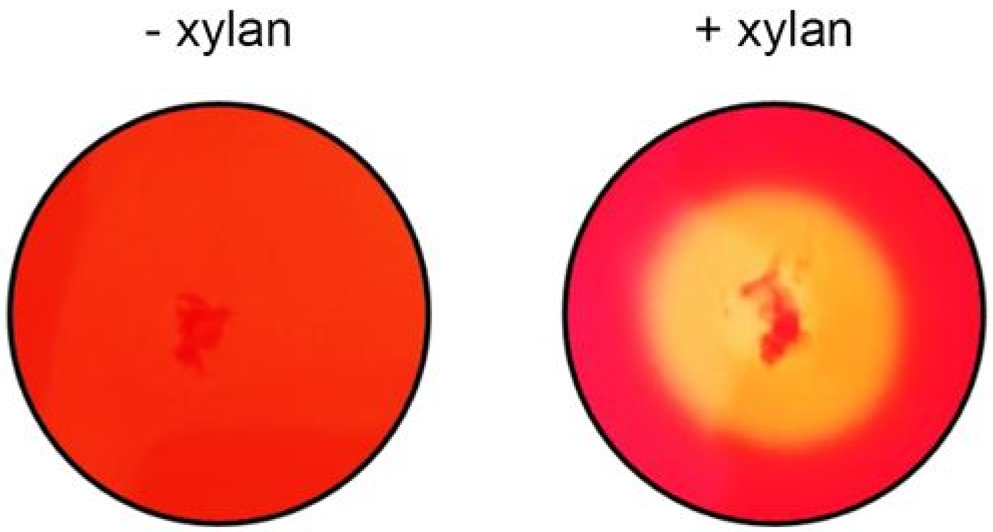

Figure S1. Clear zone on xylan agar plates after staining with Congo Red indicates the degradation of xylan as compared with control agar plate without xylan (- xylan).

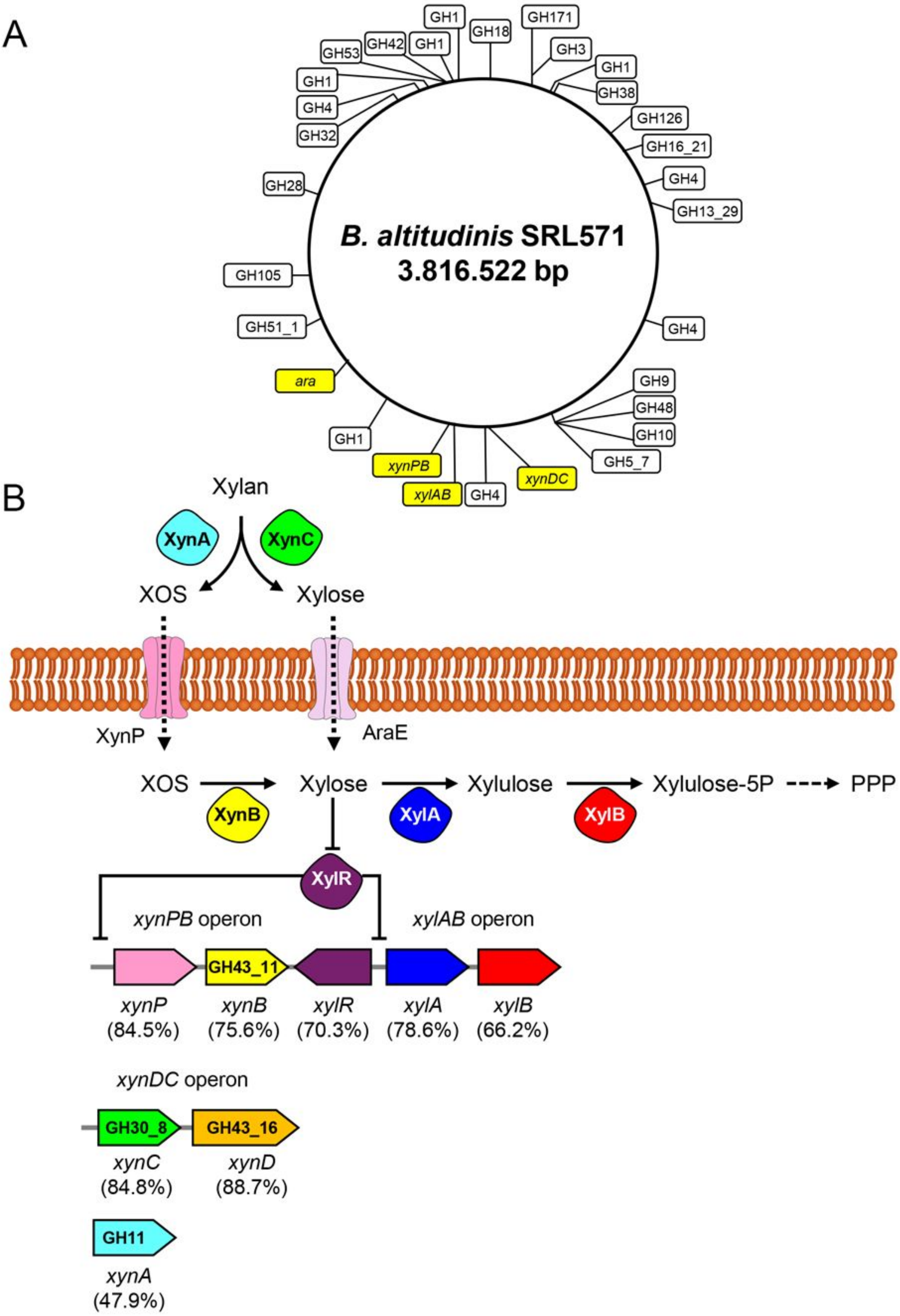

Figure S2. A) Circular representation of the *B. altitudinis* SRL571 genome, highlighting the GH annotations. The *xynDC*, *xylAB*, *xynPB,* are coloured yellow. B) A schematic representation of the xylan catabolic pathway of *B. subtilis* sp. 168. Extracellular XynA and XynC hydrolyze xylan to xylo-oligosaccharides (XOS) and xylose. XOS uptake occurs via XynP permease. Intracellular XynB hydrolyzes XOS to xylose, which is then converted to xylulose-5-phosphate by xylose isomerase and xylulose kinase. Xyl repressor (XylR in purple) controls the expression of both *xynPB* and *xylAB* operons.

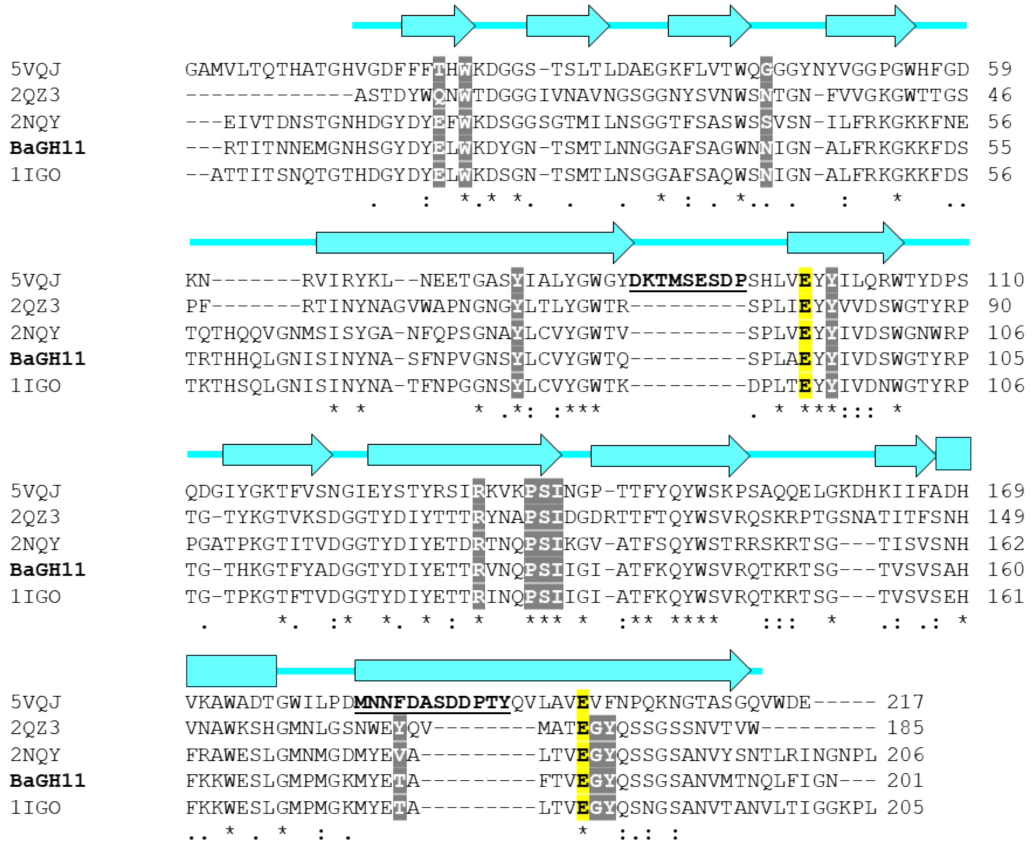

Figure S3. Structural alignment of BaGH11. The BaGH11 sequence was structurally aligned with the homologs from *Bacillus subtilis* (PDB: 2QZ3), *Bacillus* sp. (NCL 86-6-10) (PDB: 2NQY), *Bacillus subtilis* B230 (PDB: 1IGO) and from compost-derived bacterial consortia (PDB: 5VQJ). Secondary structure elements were extracted from PDB 2QZ3. Catalytic and active site residues are colored yellow and gray, respectively. The extra loops responsible for exo-activity are underlined in bold. The signal sequence of BaGH11 was removed.

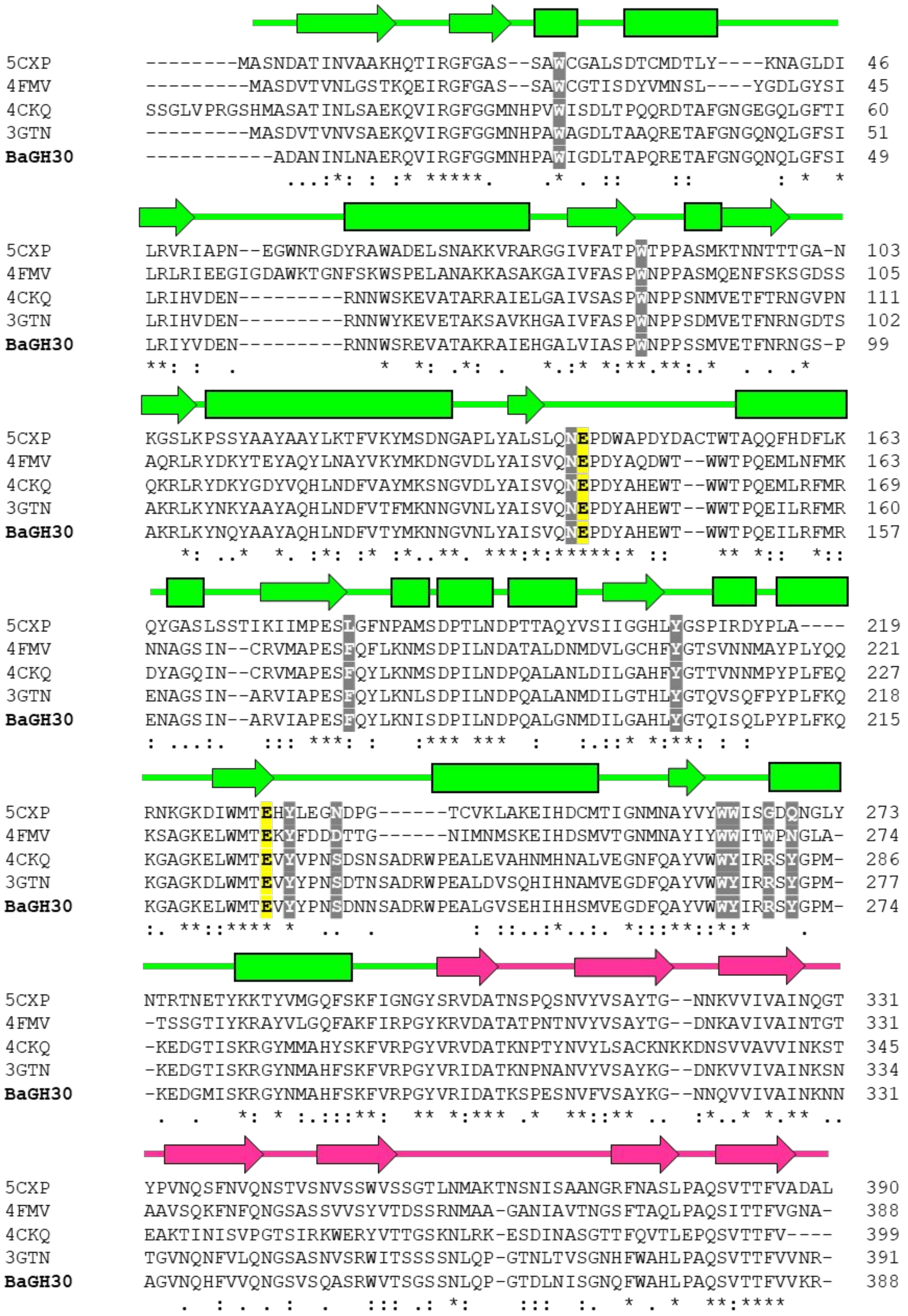

Figure S4. Structural alignment of BaGH30. The BaGH30 sequence was structurally aligned with the homologs from *Bacillus subtilis* sp. 168 (PDB: 3GTN), *Clostridium thermocellum* (PDB: 4CKQ), *Clostridium papyrosolvens* C71 (PDB: 4FMV) and from *Clostridium acetobutylicum* (PDB: 5CXP). Secondary structure elements were extracted from PDB 3GTN. Catalytic and active site residues are colored yellow and gray, respectively. The signal sequence of BaGH30 was removed.

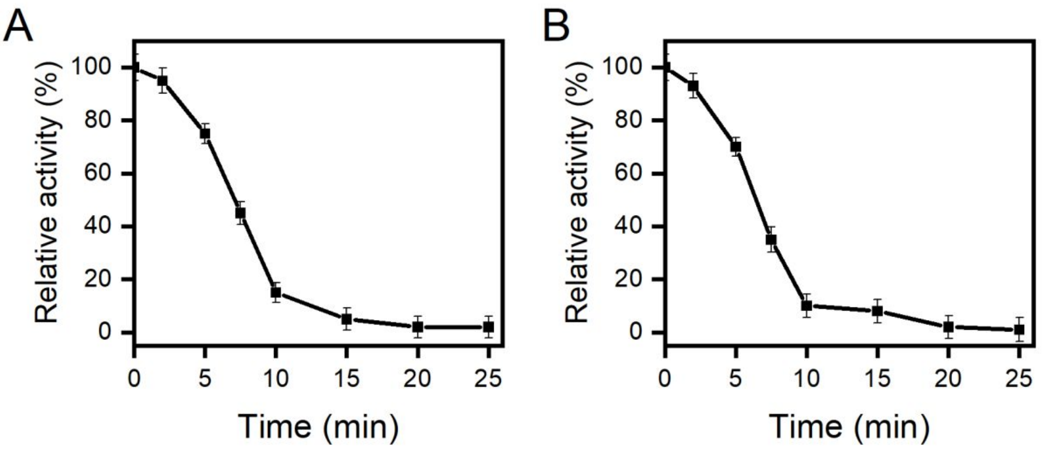

Figure S5. Thermal stability of BaGH11 and BaGH30 at T_{opt}. Thermal stability was determined by incubating BaGH11 in PB at pH 7.0 and 55 °C, and BaGH30 in PB at pH 8.0 and 60 °C. Experiments were performed in triplicate, and the error bars refer to standard deviation (n = 3).

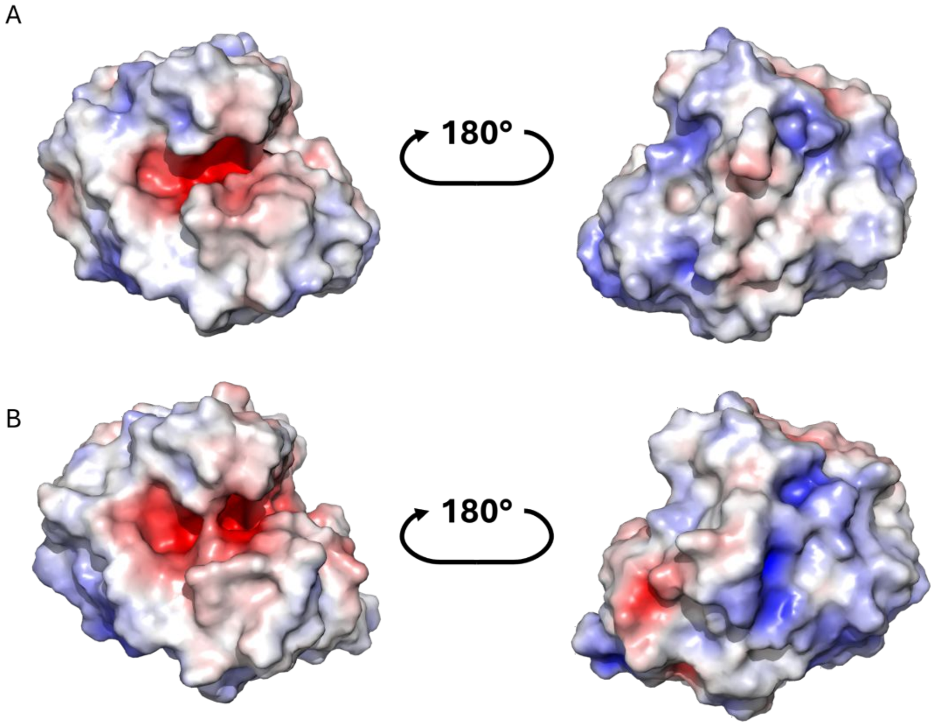

Figure S6. Surface charge of (A) BaGH11 and (B) GH11 from *Bacillus* NCL 87-6-10 (PDB: 2F6B). Electrostatic potential surfaces were calculated with APBS Electrostatics Plugin in Pymol. The red and blue colours represent negatively and positively charged amino acid residues, respectively.

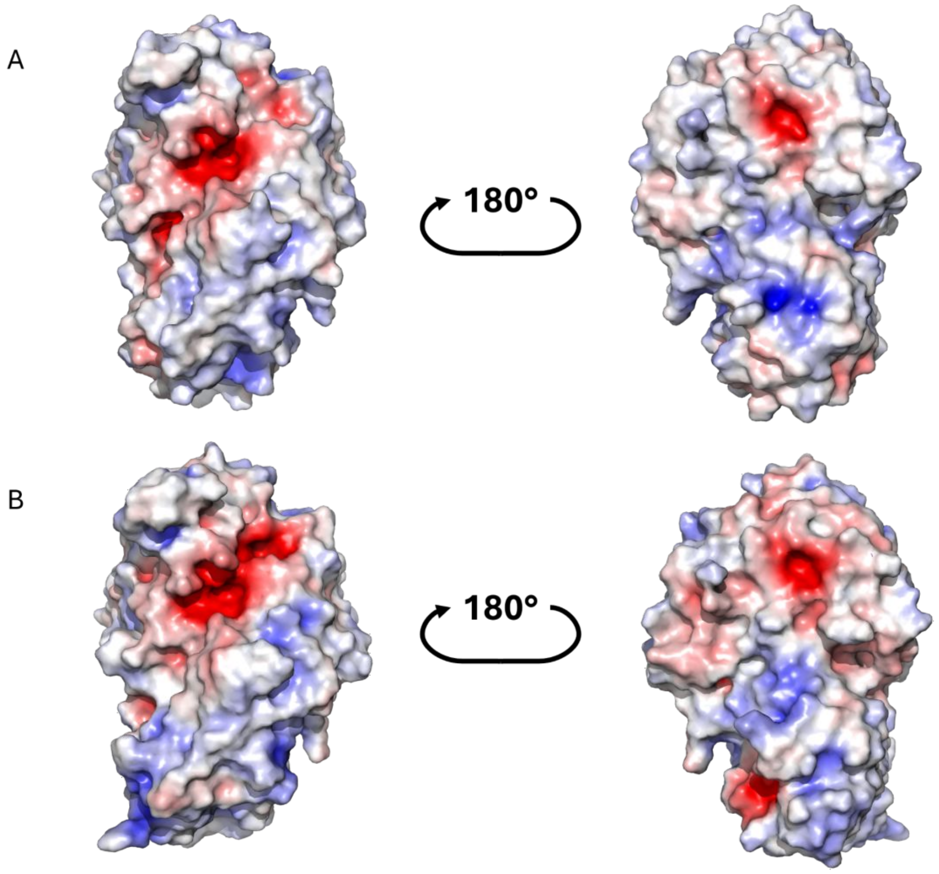

Figure S7. Surface charge of (A) BaGH30 and (B) GH30 from *Clostridium thermocellum* (PDB: 4CKQ). Electrostatic potential surfaces were calculated with APBS Electrostatics Plugin in Pymol. The red and blue colours represent negatively and positively charged amino acid residues, respectively. The net charge at pH 7.0 for BaGH30 is 3.1, while that of GH30 from *Clostridium thermocellum* is 3.8.
